# Explaining inequality tolerance in the lab: effects of political efficacy and prospects of mobility on collective demand for redistribution

**DOI:** 10.1038/s41598-023-42715-9

**Published:** 2023-09-23

**Authors:** Giannis Lois, Katerina Petkanopoulou

**Affiliations:** 1 Department of Microeconomics and Public Economics, School of Business and Economics, Maastricht University, 6200 MD Maastricht, The Netherlands; 2 Department of Psychology, Faculty of Social Sciences, University of Crete, Rethymno, Greece; 3 Department of Psychology, Panteion University of Athens, Athens, Greece

**Keywords:** Human behaviour, Psychology

## Abstract

The low public demand for redistribution despite growing economic inequality has been characterized as a paradox especially for disadvantaged individuals. One prominent explanation for people’s tolerance to growing inequality posits that increased optimism about prospects of upward mobility undermines support for redistribution. A less explored explanation postulates that low political efficacy of disadvantaged individuals to enact meaningful change erodes collective demand for redistribution. In two preregistered experiments, we create a dynamic environment where low-income individuals collectively demand income redistribution by contributing to a public pool (collective action strategy), compete with each other for high-income group positions (individual mobility strategy), or avoid risks and disengage from both strategies (social inaction strategy). Lack of political efficacy, operationalized as high redistribution thresholds, gradually curtailed collective action, while exposure to high prospects of mobility did not influence collective action even when income group boundaries were highly permeable. Across participants, we identified three behavioral types (i.e., “mobility seekers”, “egalitarians”, and “disillusioned”) whose prevalence was affected by political efficacy but not by prospects of mobility or actual group permeability. These results cast doubt on the universality of the prospects of mobility hypothesis and highlight the prominent role of political inequality in the perpetuation of economic inequality.

## Introduction

Growing economic inequality worldwide is characterized as one of the main challenges of our time with pervasive consequences for social cohesion, growth, and health outcomes^1,2^. Despite the rampant inequality, people often tolerate and in certain cases even support low levels of redistribution^3,4^. This divergence between growing inequality and low support for redistribution has been characterized as a paradox for median voters^5^ and even more so for low socioeconomic status individuals who are harmed by the unequal distribution of resources.

In this article, we use a novel experimental paradigm to investigate two alternative explanations for people’s tolerance to inequality. The most prominent explanation focuses on ideology and posits that optimism about personal prospects of upward mobility reduces support for redistribution^6–8^. A less explored explanation focuses on political factors that render disadvantaged individuals’ collective efforts to reduce economic inequality inefficient and therefore undermine their willingness to demand redistribution.

### Prospects of mobility hypothesis

Evidence from national and cross-national surveys shows that high prospects of mobility go hand in hand with high inequality tolerance and low public support for redistribution^7,9–12^. Experimental evidence corroborates this hypothesis by showing that exposure to high prospects of mobility increases tolerance to inequality^9,12^. Intriguingly, more unequal societies are characterized by lower inter-generational mobility rates^13,14^ suggesting that optimistic prospects of mobility in these societies may result in the perpetuation of high economic inequality.

Despite its prominence, this hypothesis raises questions about how individuals maintain optimistic views about their prospects of mobility in less socially mobile societies. One plausible explanation is that modern meritocratic societies emphasize internal attributions of failure (i.e., incompetent poor) and personal success stories thus masking the relatively impermeable boundaries between socioeconomic groups^3,15^. In this system of tokenism, people often endorse meritocratic beliefs and strive for upward mobility^16^. This explanation is consistent with a system justifying account, which posits that people, including low-status individuals, are motivated to justify the existing social and economic system to satisfy basic psychological needs^17,18^.

Notwithstanding these ideological explanations, recent work has found that the divergence between high prospects of mobility and low actual social mobility rates predominantly characterizes the US^7,19^, but not other countries^9^. Furthermore, recent survey data suggest that negative or stagnating personal mobility experiences lead to a downward adjustment of personal prospects of mobility^20^ thus questioning the persistence of optimistic prospects of mobility in less socially mobile societies. In light of these critical points, we turned our attention to political factors that could explain the tolerance to growing economic inequality.

### Lack of political efficacy hypothesis

Evidence from many countries worldwide suggests that governments respond primarily to the preferences of wealthy citizens and much less to the preferences of less affluent groups^21–23^. This imbalance of political power is further strengthened when economic inequality is high as wealthy citizens have more power to influence public policies^24,25^. The low political efficacy of disadvantaged individuals may lead to low electoral turnout, eroding public confidence in democratic institutions, and a growing trend of political inaction^26,27^.

Based on these insights, we propose an alternative explanation in which disadvantaged individuals lack the political efficacy to enact meaningful change through voting or other forms of political participation^21–23^. Therefore, instead of a collective reaction to unfair and high levels of inequality, disadvantaged individuals strive for individual mobility within this unfair system^28^ or become disillusioned and passive citizens^26^. According to system justification theory, such lack of political power enhances disadvantaged individuals’ motivation to justify the system and legitimize economic inequality^29^. However, other work suggests that despite the low political efficacy and the resulting political inaction, disadvantaged individuals still recognize the unfairness of wealth distribution^30,31^ and the limited chances for social mobility^9^.

### Social identity theory and reactions to inequality

Both the ideological and the political explanation of the tolerance to growing inequality are consistent with social identity models that consider group efficacy beliefs and perceived permeability of group boundaries as determinants of disadvantaged group members’ responses to social inequality^8,32^. Surveys and experimental studies have shown that permeable or semi-permeable (compared to strictly impermeable) group boundaries, and perceptions thereof, increase the willingness of low-status members (e.g., ethnic minorities, women, national groups) to engage in individual strategies^33–36^. On the other hand, perceived group efficacy is associated with collective action against inequality^37,38^.

### Current research

Our research builds upon and extends this work by examining whether sociostructural characteristics (i.e., lack of political efficacy and permeability of income group boundaries) and perceptions thereof (i.e., high prospects of mobility) undermine collective reactions to economic inequality. Unlike previous studies that employed self-report measures, we develop a novel economic experiment where low-income individuals make costly decisions in the face of large and unfair economic inequality. In modern meritocratic societies, access to high-status groups is restricted and individuals compete against each other to achieve upward mobility. In this competitive setting, demanding income redistribution through various forms of collective action may entail financial, reputational, or opportunity costs^39^. Even voting for political parties that advocate for redistribution can be costly when these parties’ agenda conflicts with voters’ preferences in other social or economic issues.

Based on this insight, we conceptualize individual mobility and collective demand for redistribution as competing costly strategies. We use a repeated threshold public goods game where low-income individuals can collectively reduce intergroup inequality by reaching predetermined redistribution thresholds. At the same time, they compete with each other for upward mobility after being exposed to information about their prospects of moving to a high-income position. Given that both collective action against inequality and striving for individual mobility entail risks, individuals may opt for a risk-free strategy (social inaction), especially when they perceive low political efficacy and no prospects of mobility. We thus introduce a third social inaction strategy which accounts for the possibility of political passivity^26^ and eliminates the strict negative interdependence between income redistribution and individual mobility strategies. In other words, the presence of a third risk-free strategy allows us to exclude the possibility that investment in one strategy (e.g., individual mobility) merely reflects disbelief in the other strategy (e.g., collective action).

In light of the discrepancy between optimistic prospects of mobility and low actual social mobility rates that characterize many societies^13,14^, we also investigate how repeated frustration of high prospects of mobility influences the strategies that people adopt to navigate economic inequalities. Lastly, we explore the dynamic evolution of public demand for redistribution in the presence of previous successful or failed attempts to collectively reduce inequality. Our dynamic setting allows the identification of different behavioral types based on how disadvantaged individuals’ reactions to inequality evolve over time.

## Study 1

In Study 1, we assessed to what extent lack of political efficacy and exposure to high prospects of mobility undermine low-income individuals’ demand for redistribution over time. In this study, income group boundaries were strictly impermeable and thus personal mobility prospects were repeatedly frustrated. To emulate the real-life ambiguity regarding the permeability of group boundaries^43^, disadvantaged individuals received ambiguous feedback about the reasons for the absence of upward mobility (i.e., insufficient personal investment in this strategy or impermeable group boundaries).

Consistent with aforementioned social identity models^32^ and the well-documented gradual erosion of the public good^44^, we hypothesized that lack of political efficacy gradually undermines collective action against inequality as disadvantaged individuals cease demanding redistribution after repeated failure to achieve it (H1). Given that people update their prospects of mobility based on evidence about actual mobility rates^9,20^, we hypothesized that exposure to high prospects of mobility increases investment in individual mobility (H2) but this prospects-of-mobility effect gradually declines as group boundaries are actually impermeable (H3a).

### Methods

#### Participants and general procedure

Participants were UK residents and were recruited through the online platform Prolific Academic. We recruited 232 participants (*Μ*_age_ = 31.1, *SD* = 10.7, 149 females). We used a 2 × 2x10 mixed design whereby participants were randomly assigned to a high or a low efficacy condition and a high or a low prospects-of-mobility condition (see Table S1 for demographics). Participants played the threshold public goods game for ten rounds. Experimental sessions were conducted online using the software o-Tree^45^. Four participants took part simultaneously in each session and they interacted anonymously online. Upon entering the session, participants read detailed instructions about the threshold public goods game, and then answered four comprehension questions (see Supporting Information S1 for instructions). The experiment lasted around 25 min. At the end of the session, one round was randomly selected to determine participants’ bonus payment. We obtained informed consent from all participants prior to taking part in the experiment and after debriefing at the end of the session. No observations were excluded from the analysis.

Study design, sample sizes, hypotheses, and statistical analyses were preregistered and are available at https://osf.io/zv34k (Study 1), https://osf.io/zsudy/, and https://osf.io/sg3rn (Study 2). Some preregistered hypotheses are not presented here to increase clarity and focus on the main objectives of the present work. All preregistered hypotheses are reported and tested in the Supporting Information S1. We conducted a priori power analysis to obtain 0.80 power for main effects and two-way interactions to detect a medium effect size of η_p_^2^ = 0.06 at α = 0.05 after correcting for the number of hypotheses (Bonferroni correction). The analyses regarding the behavioral types were not preregistered. Both studies were carried out in accordance with relevant guidelines and regulations and were approved by the Ethical Review Committee Inner City Faculties of Maastricht University.

#### Experimental paradigm

**Figure 1:**
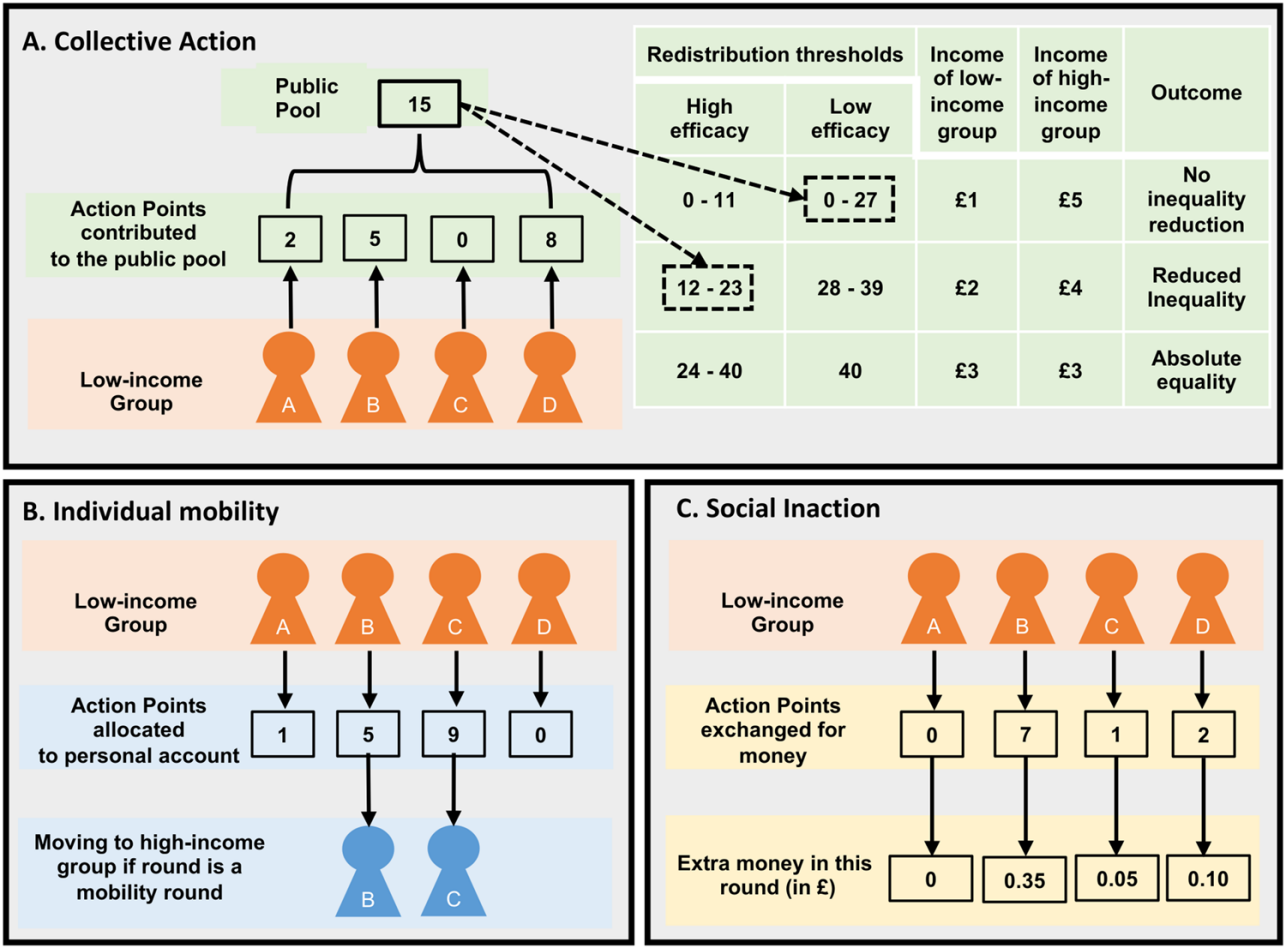
Hypothetical example of how the four low-income members invest their APs in the three strategies in a given round. Depending on contributions to the public pool, three different redistribution outcomes are possible: no inequality reduction, reduced inequality, absolute equality (**A**). In this example, the four low-income members contributed 15 APs to the public pool, which results in no inequality reduction in the low efficacy condition and reduced inequality in the high efficacy condition. Low-income members can also pursue an individual mobility strategy by allocating their APs to a personal account (**B**). In this example, participants B and C allocated the most APs to their personal account. In a “mobility round”, these two participants would move to the high-income group. Low-income members can also opt for a risk-free social inaction strategy by exchanging their APs for money (**C**).

At the beginning of each session, participants performed a Counting Zeros Task^46^ to produce their income. This task consisted of counting, within two minutes, the number of zeros that are contained in 5 × 5 tables of numbers. Participants who correctly counted the zeros in a predetermined (but unknown to them) number of tables, successfully produced their income and participated in the next experimental phase. Subsequently, we instructed participants that they will be randomly assigned to a low-income (£1) or a high-income group (£5). In reality, the four participants of each session were assigned to the low-income group (i.e., deception), which is the group whose reactions to inequality we aim to investigate. After group assignment, the four low-income members were given ten independent opportunities (i.e., ten rounds) to increase their income by playing the threshold public goods game. In each round, they were endowed with ten Action Points (APs) which they could invest in three different strategies (i.e., three main dependent variables):

(1) Collective action strategy (Fig. 1A): low-income members could collectively demand income redistribution by contributing their APs to the public pool. In each round, if the total contributions to the public pool exceeded certain thresholds, income was redistributed from the high-income to the low-income group. We imposed a “reduced inequality” and an “absolute equality” redistribution threshold. When contributions exceeded only the former threshold, income redistribution led to reduced intergroup inequality (low-income: £2 and high-income: £4). When contributions exceeded both thresholds, income redistribution led to equal incomes between the two groups (low-income: £3 and high-income: £3). (2) Individual mobility strategy (Fig. 1B): low-income members could increase their chances of moving to the high-income group by allocating their APs to a personal account. Participants were informed that with some probability each of the ten rounds could be a “mobility round”. In a “mobility round”, two out of the four low-income members, those who allocated the most APs to their personal account, move to the high-income group and thus earn a high income for this specific round. (3) Social inaction strategy (Fig. 1C): low-income members could avoid risks related to collective action or individual mobility by exchanging their APs for money (1AP = £0.05).

To operationalize political efficacy, we changed the redistribution thresholds. In the low efficacy condition, the redistribution thresholds were higher than the high efficacy condition and thus participants needed to contribute more APs to the public pool to reach these thresholds. To manipulate prospects of mobility, we informed participants and we reminded them at the end of each round, that low-income members of previous sessions stated that each round is a “mobility round” with 10% (low prospects of mobility) or 50% (high prospects of mobility) probability. This information was truthful in both conditions as there were indeed participants that indicated probabilities close to 10% and 50%, respectively.

Participants were informed that the outcome of one round (i.e., successful collective action or upward mobility) affects their income and their group membership in this round but not in subsequent rounds. At the start of each round, participants are members of the low-income group and are endowed with £1. Thus, each round is independent. At the end of each round, participants received real feedback about whether collective action was successful and how this affected the group’s income in this specific round. In both high and low prospects-of-mobility conditions, participants were also informed that they failed to move to the high-income group (i.e., impermeable system) for one of the following reasons: (1) boundaries between income groups were impermeable (i.e., no mobility round) or (2) participant exerted insufficient personal effort/investment (i.e., few APs in personal account).

At the end of the first and last round and before they receive any feedback, we elicited participants’ beliefs regarding the fairness of the initial income gap, the permeability of group boundaries, and the efficacy of the low-income group. At the end of the experiment, we also assessed the extent to which participants identify with the low-income group. All aforementioned beliefs were measured on 7-point Likert scales. At the end of the experimental session, participants were debriefed about the purpose of the study and the fact that all participants were assigned to the low-income group (i.e., no random group assignment).

#### Data analyses

To test the effect of efficacy and prospects of mobility on the time evolution of investment in the three strategies, we sequentially fitted fixed-effect models and linear mixed effect models. The random intercept and random slope model provided the best model fit. Hence, all reported results are based on this model. We performed separate three-way mixed ANOVAs to quantify how investment in each of the three strategies differs across conditions and over time (Tables 1 and S10 for robustness tests). Thus, we adjusted statistical significance for three comparisons (Bonferroni correction: *α* = 0.017). Similar analysis was performed to quantify how perceptions of fairness, permeability, and efficacy differ across efficacy and prospects-of-mobility conditions and over time (Fig. S1 and Table S4).

**Table 1:** Three-way mixed ANOVA testing the effects of efficacy, prospects of mobility, and time on investment in three strategies.

Predictor variables	df	Outcome variables								
		Collective action			Individual mobility			Social inaction		
		F	p	η^2^	F	p	η^2^	F	p	η^2^
Efficacy	1, 228	3.00	0.085	0.010	0.58	0.447	0.003	0.65	0.422	0.003
Prospects of mobility	1, 228	0.63	0.425	0.003	6.93*	0.009	0.030	3.21	0.075	0.010
Time	1, 228	13.90*	< 0.001	0.060	2.29	0.131	0.010	4.92	0.027	0.020
Efficacy × prospects of mobility	1, 228	0.12	0.728	0.001	0.12	0.728	0.001	0.09	0.764	< 0.001
Efficacy × time	1, 228	35.34*	< 0.001	0.130	10.94*	0.001	0.050	5.79*	0.017	0.020
Prospects of mobility × time	1, 228	0.14	0.706	0.001	0.15	0.694	0.001	0.12	0.729	0.001
Efficacy × prospects of mobility × time	1, 228	0.15	0.701	0.001	0.51	0.477	0.002	0.73	0.394	0.003

*p < 0.017 (p value adjusted for multiple testing).

### Results

As depicted in Table 1, we did not observe any significant interaction between efficacy and prospects of mobility on investment in any of the three strategies indicating that efficacy and prospects of mobility exert independent effects.

#### Efficacy effects

**Figure 2:**
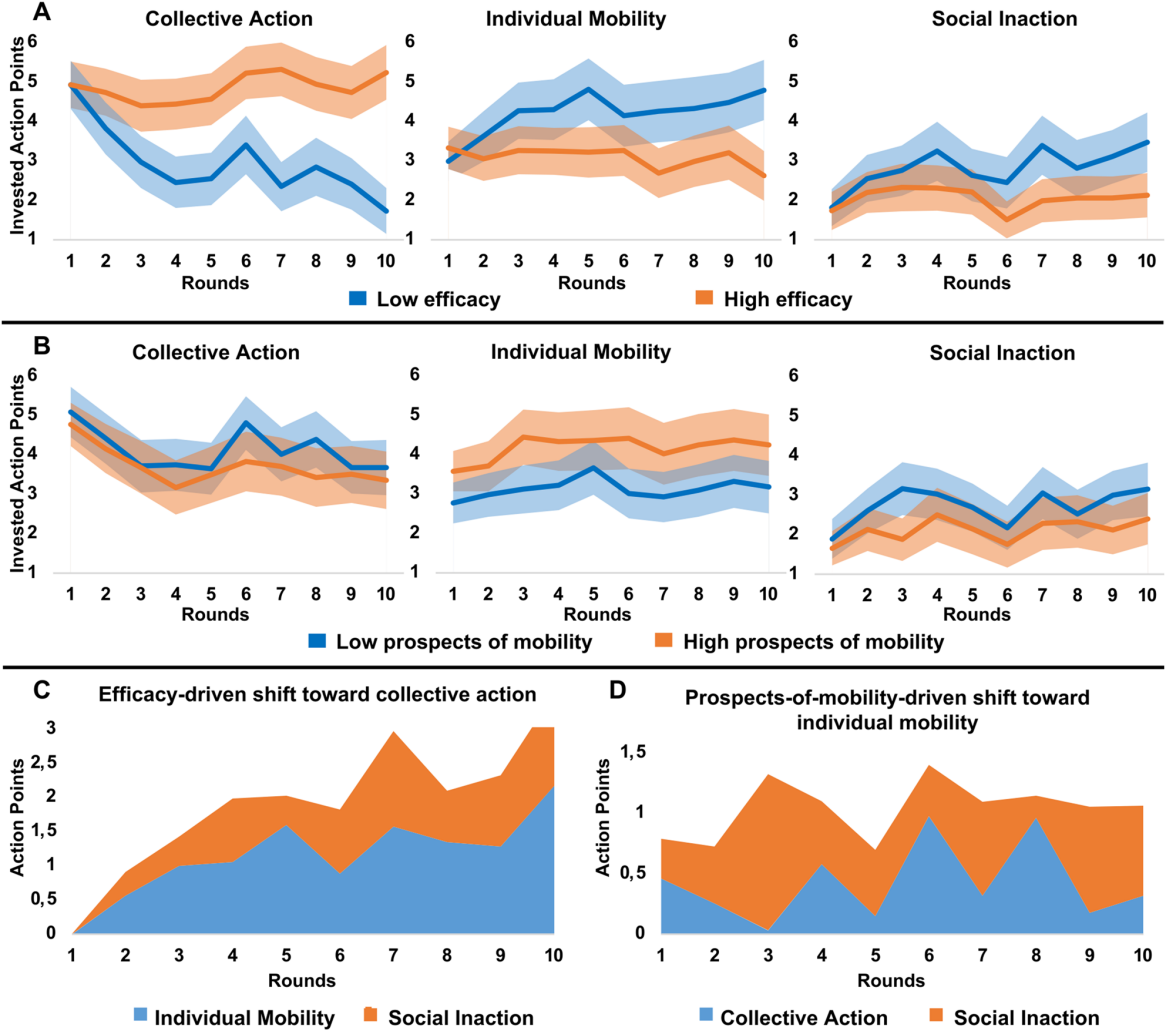
Time evolution of average investment in three strategies across efficacy (**A**) and prospects of mobility (**B**) conditions. Bands around the averages in A and B indicate the 95% confidence interval of the mean. Action Points shifted from individual mobility (blue) or from social inaction (orange) to collective action in the high vs low efficacy condition (**C**). Action Points shifted from collective action (blue) or from social inaction (orange) to individual mobility in the high vs low prospects-of-mobility condition (**D**).

Consistent with H1, low (vs high) efficacy of disadvantaged individuals gradually curtailed collective demand for redistribution (*F*(1, 228) = 35.34, *p* < 0.001, η^2^ = 0.130). This efficacy by time interaction effect was driven by the declining popularity of the collective action strategy in the low efficacy condition (*F*(1, 113) = 52.40, *p* < 0.001, η^2^ = 0.320). In both efficacy conditions, participants invested around half of their APs in redistribution in the first round. However, by the last round, the share of APs invested in redistribution had fallen to 17% in the low efficacy condition, while it remained stable around 50% in the high efficacy condition (Fig. 2A). We also observed weaker efficacy by time interaction effects on investment in individual mobility (*F*(1, 228) = 10.94, *p* = 0.001, η^2^ = 0.050) and social inaction (*F*(1, 228) = 5.79, *p* = 0.017, η^2^ = 0.020). Overall, the low efficacy-driven decline in collective action was associated with growing popularity of individual mobility (59%) and social inaction (41%) strategies (Fig. 2C).

#### Prospects-of-mobility effects

Contrary to the prospects of mobility hypothesis, exposure to high (vs low) prospects of mobility had no impact on collective action against inequality, but, as hypothesized (H2), it led to increased investment in individual mobility (*F*(1, 228) = 6.93, *p* = 0.009, η^2^ = 0.030). Contrary to H3a, the prospects-of-mobility effect on individual mobility did not decline over time as investment in this strategy remained stable in both prospects-of-mobility conditions (Fig. 2B). The persistent pursuit of individual mobility across the ten rounds indicates that participants were not discouraged by the repeated frustration of their mobility prospects. Exposure to high (vs low) prospects of mobility did not affect investment in social inaction. Overall, the prospects-of-mobility-driven rise in the popularity of individual mobility was associated with reduced investment in both social inaction (60%) and collective action (40%) (Fig. 2D).

#### Effects on beliefs

Overall, the income gap between the two groups was overwhelmingly perceived as unfair across all conditions (Fig. S1). Low efficacy and exposure to high prospects of mobility had no impact on fairness perceptions (Table S4). As expected, exposure to high prospects of mobility led to increased perceptions of group permeability (*F*(1, 228) = 19.50, *p* = 0.016, η^2^ = 0.080). However, participants in both prospects-of-mobility conditions updated downwards their group permeability perceptions over time (*F*(1, 228) = 127.13, *p* < 0.001, η^2^ = 0.360). As expected, perceived group efficacy was lower in the low efficacy condition (*F*(1, 228) = 37.50, *p* < 0.001, η^2^ = 0.140) and this efficacy effect increased over time (*F*(1, 228) = 50.48, *p* < 0.001, η^2^ = 0.180).

## Study 2

In Study 1, access to the high-income group was systematically blocked. However, in real life, group boundaries are rarely strictly impermeable. Thus, in Study 2, disadvantaged individuals were again exposed to high or low prospects of mobility but now they personally experienced or witnessed upward mobility in the first round (low group permeability condition).

Study 2 aims to replicate the efficacy by time interaction effect on collective action (H1) and the main prospects-of-mobility effect on individual mobility (H2) that we observed in Study 1. However, unlike Study 1 where participants never experienced upward mobility, we hypothesized that the prospects-of-mobility effect on individual mobility increases over time (H3b) after an early (i.e., first round) experience of upward mobility. Specifically, exposure to high prospects of mobility in this context may lead to sustained investment in individual mobility despite the absence of upward mobility in subsequent rounds. On the other hand, exposure to low prospects of mobility after an early experience of upward mobility may lead to disengagement from this strategy in subsequent rounds.

In Study 2, we also assessed disadvantaged individuals’ reactions to inequality when income group boundaries were indeed highly permeable. Thus, we also introduced a high group permeability condition where prospects of mobility were frequently realized. We hypothesized that high, compared to low, permeability of group boundaries gradually undermines collective action against inequality (H4) and increases investment in individual mobility (H5).

### Methods

#### Participants and general procedure

Participants were UK residents and were recruited through the online platform Prolific Academic. We recruited 664 participants (*Μ*_age_ = 32.0, *SD* = 10.0, 265 females). We used a 2 × 3x10 mixed design whereby participants were randomly assigned to a low or a high efficacy condition and a low prospects-of-mobility & low permeability, a high prospects-of-mobility and low permeability or a high prospects-of-mobility & high permeability condition (see Table S1 for demographics). We analyzed participants’ investment behavior across ten rounds. The experimental procedure was identical to Study 1.

#### Experimental paradigm

Similar to Study 1, we used a repeated threshold public goods game. Study 2 differed from Study 1 in that we introduced one or multiple ”mobility rounds” and we eliminated the ambiguity regarding the permeability of group boundaries. Participants received explicit feedback about mobility outcomes. Participants who kept track of the mobility outcome in each round could thus infer that group boundaries were permeable only in the first round. We introduced two low permeability conditions where participants were exposed to either low or high prospects of mobility. In both low permeability conditions, upward mobility took place only in the first round. In subsequent rounds, group boundaries were impermeable. In a third condition (high prospects-of-mobility & high permeability), mobility took place in the first round and in four of the nine subsequent rounds. In “mobility rounds”, two out of the four low-income members personally experienced upward mobility while the other two low-income members witnessed it (i.e., vicarious experience). In the remaining rounds, participants received explicit feedback that no low-income member achieved upward mobility. We did not differentiate between personal and vicarious experiences of upward mobility as both groups displayed similar patterns of investment (see Table S14). As in Study 1, we elicited participants’ beliefs regarding the fairness of the income gap, the group permeability, and the group efficacy.

#### Data analyses

Similar to Study 1, we used the random intercept and random slope model, which provided the best fit to the behavioral data. We performed separate three-way mixed ANOVAs to quantify the effect of efficacy, prospects of mobility, and group permeability on the time evolution of investment in the three strategies (Tables 2 and S11 for robustness tests). To test H2 and H3b, we compared exposure to low vs high prospects of mobility in a low group permeability context (Test 1). To test H4 and H5, we compared high vs low group permeability after exposure to high prospects of mobility (Test 2). Since two separate tests were performed for each of the three strategies, we adjusted statistical significance for six comparisons (Bonferroni correction: α = 0.008). Similar analyses were performed to quantify efficacy, prospects-of-mobility, and group permeability effects on perceptions of fairness, group permeability and group efficacy (Fig. S1 and Table S5).

**Table 2:** Three-way mixed ANOVA testing the effects of efficacy, prospects of mobility, group permeability, and time on investment in three strategies.

Predictor variables	df	Outcome variables								
		Collective action			Individual mobility			Social inaction		
		F	p	η^2^	F	p	η^2^	F	p	η^2^
Efficacy^1^	1, 439	13.17*	< 0.001	0.030	10.82*	0.001	0.020	0.88	0.347	0.002
Prospects of mobility^1^	1, 439	2.49	0.115	0.006	24.57*	< 0.001	0.050	9.59*	0.002	0.020
Time^1^	1, 439	147.90*	< 0.001	0.250	18.49*	< 0.001	0.040	237.44*	< 0.001	0.350
Efficacy × prospects of mobility^1^	1, 439	0.12	0.726	< 0.001	< 0.01	0.936	< 0.001	0.10	0.750	< 0.001
Efficacy × time^1^	1, 439	21.81*	< 0.001	0.050	3.45	0.064	0.008	37.36*	< 0.001	0.080
Prospects of mobility × time^1^	1, 439	0.61	0.436	0.001	0.10	0.745	< 0.001	1.09	0.297	0.002
Efficacy × prospects of mobility × time^1^	1, 439	1.16	0.691	< 0.001	1.22	0.269	0.003	2.05	0.153	0.005
Efficacy^2^	1, 437	10.47*	0.001	0.020	2.25	0.135	0.005	5.88	0.016	0.010
Permeability^2^	1, 437	0.24	0.626	0.001	1.58	0.209	0.004	0.24	0.624	0.001
Time^2^	1, 437	174.56*	< 0.001	0.290	< 0.01	0.989	< 0.001	164.95*	< 0.001	0.270
Efficacy × permeability^2^	1, 437	0.65	0.421	0.001	2.75	0.098	0.006	1.26	0.262	0.003
Efficacy × time^2^	1, 437	44.27*	< 0.001	0.090	1.41	0.236	0.003	23.85*	< 0.001	0.050
Permeability × time^2^	1, 437	0.07	0.792	< 0.001	21.76*	< 0.001	0.050	22.31*	< 0.001	0.050
Efficacy × permeability × time^2^	1, 437	4.85	0.028	0.010	3.77	0.053	0.009	0.01	0.910	0.003

^1^Test 1: ANOVA testing the effects of efficacy and prospects of mobility (only low permeability conditions were included).

^2^Test 2: ANOVA testing the effects of efficacy and group permeability (only high prospects-of-mobility conditions were included).

*p < 0.008 (p value adjusted for multiple testing).

### Results

As shown in Table 2, Test 1 and Test 2 yielded similar efficacy effects and revealed no interaction between efficacy and prospects of mobility (Test 1) or efficacy and group permeability (Test 2). This pattern suggests that efficacy effects are robust and independent from prospects-of-mobility and group permeability manipulations. Thus, in the text below, we describe efficacy effects in the entire sample (i.e., all three prospects-of-mobility & group permeability conditions).

#### Efficacy effects

**Figure 3:**
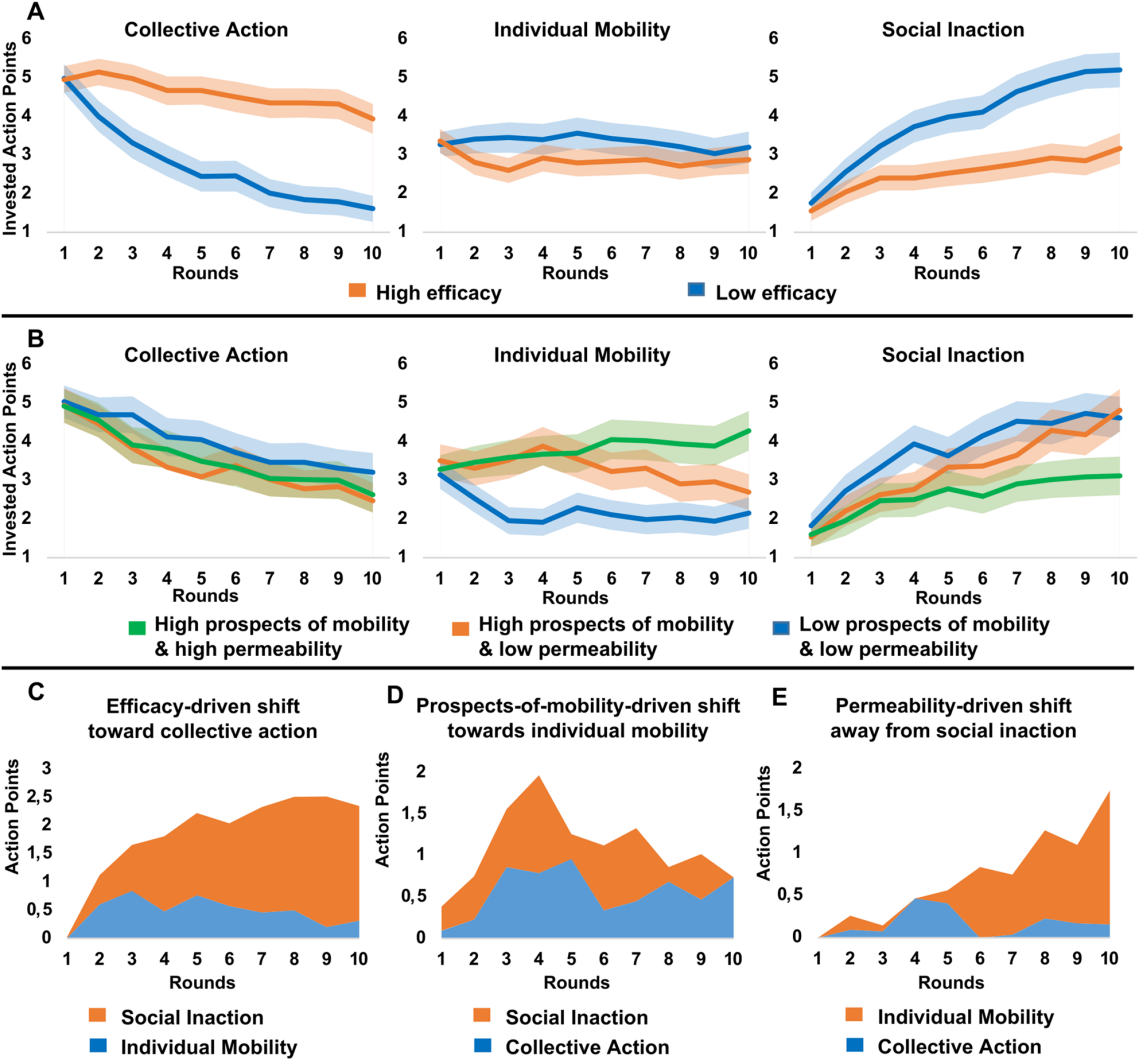
Time evolution of average investment in three strategies across efficacy (**A**), prospects of mobility (**B**), and group permeability (**B**) conditions. Bands around the averages in A and B indicate the 95% confidence interval of the mean. Action Points shifted from individual mobility (blue) or from social inaction (orange) to collective action in the high vs low efficacy condition (**C**). Action Points shifted from collective action (blue) or from social inaction (orange) to individual mobility in the high vs low prospects-of-mobility condition (**D**). Action Points shifted from social inaction to collective action (blue) or individual mobility (orange) in the high vs low group permeability condition (**E**).

Similar to Study 1 and consistent with H1, low (vs high) efficacy gradually undermined collective demand for redistribution (*F*(1, 662) = 53.92, *p* < 0.001, η^2^ = 0.080). In both efficacy conditions, low-income individuals invested half of their APs in collective action in the first round, but investment in collective action declined over time (Fig. 3A). Although this gradual decline was steeper when efficacy was low, it was significant in both low (*F*(1, 331) = 216.22, *p* < 0.001, η^2^ = 0.400) and high (*F*(1, 331) = 36.22, *p* < 0.001, η^2^ = 0.100) efficacy conditions. By the last round, investment in redistribution had declined to 20% in the low efficacy condition and to 37% in the high efficacy condition.

In both efficacy conditions, participants gradually shifted away from collective action mostly to social inaction, which gradually became the most popular strategy. However, this shift was more prominent in the low (compared to high) efficacy condition (*F*(1, 662) = 50.17, *p* < 0.001, η^2^ = 0.070). In fact, 76% of the low efficacy-driven decline in collective action was associated with increased investment in social inaction (Fig. 3C). Notably, efficacy had only a marginal main effect on individual mobility (*F*(1, 662) = 6.36, *p* = 0.009, η^2^ = 0.010).

#### Prospects-of-mobility effects in low group permeability context

Contrary to the prospects of mobility hypothesis but similar to Study 1, exposure to high (vs low) prospects of mobility when group permeability was low did not influence collective action against inequality. Consistent with H2, exposure to high (vs low) prospects of mobility increased investment in individual mobility (*F*(1, 439) = 24.57, *p* < 0.001, η^2^ = 0.050). Contrary to H3b, we observed no prospects of mobility by time interaction effect. However, unlike Study 1 where investment in individual mobility remained stable across rounds, here we observed drastic changes in the popularity of this strategy over time (Fig. 3B). Following the experience of upward mobility in the first round, exposure to low prospects of mobility led to a steep decline in the popularity of individual mobility in the next three rounds, which remained low in the remaining seven rounds. On the other hand, exposure to high prospects of mobility slightly increased investment in individual mobility in the first four rounds but the popularity of this strategy gradually declined in subsequent rounds as repeated failure to achieve upward mobility was explicitly attributed to impermeable group boundaries.

We conducted an exploratory analysis to assess whether these different short-term and long-term trends are significant. Consistent with H3b, the prospects-of-mobility effect on investment in individual mobility gradually increased in the first four rounds (*F*(1, 439) = 23.70, *p* < 0.001, η^2^ = 0.050). However, consistent with H3a, the prospects-of-mobility effect on individual mobility gradually declined in the subsequent six rounds (*F*(1, 439) = 9.35, *p* = 0.002, η^2^ = 0.020). Overall, the prospects-of-mobility-driven increase in individual mobility was associated with both reduced investment in social inaction (52%) and reduced collective action (48%; Fig. 3D). This shift is also reflected in the lower popularity of the social inaction strategy after exposure to high prospects of mobility (*F*(1, 439) = 9.59, *p* = 0.002, η^2^ = 0.020).

#### Group permeability effects after exposure to high prospects of mobility

Contrary to H4, high (vs low) group permeability had no impact on collective action (Fig. 3B) but, consistent with H5, it led to a slow (after the fifth round) and gradual increase in the popularity of individual mobility (*F*(1, 437) = 21.76, *p* < 0.001, η^2^ = 0.050). Post hoc tests revealed that this effect was driven by both growing popularity of this strategy in the high group permeability condition (*F*(1, 220) = 11.81, *p* = 0.001, η^2^ = 0.050) and declining popularity in the low group permeability condition (*F*(1, 219) = 10.10, *p* = 0.002, η^2^ = 0.040). Low (vs high) group permeability also led to a steeper rise in the popularity of social inaction (*F*(1, 437) = 22.31, *p* < 0.001, η^2^ = 0.050). As shown in Fig. 3E, the permeability-driven decline in social inaction was predominantly associated with increased investment in individual mobility (75%).

#### Effects on beliefs

As in Study 1, the income gap was overwhelmingly perceived as unfair across all conditions (Fig. S1). Low efficacy led to decreased fairness perceptions only in the last round (*F*(1,660) = 27.22, *p* < 0.001, η^2^ = 0.040). Exposure to high prospects of mobility and high group permeability had no impact on fairness perceptions (Table S5). Perceptions of group permeability were more prevalent in the high (vs low) prospects-of-mobility condition only in the first round (*F*(1, 437) = 10.26, *p* = 0.001, η^2^ = 0.020). On the other hand, high (vs low) group permeability increased perceptions of group permeability only in the last round (*F*(1, 437) = 99.24, *p* < 0.001, η^2^ = 0.160). As expected, group efficacy perceptions were lower in the low efficacy condition (*F*(1, 660) = 81.80, *p* < 0.001, η^2^ = 0.080) and this efficacy effect increased over time (*F*(1, 660) = 70.43, *p* < 0.001, η^2^ = 0.060).

## Behavioral types

Apart from average investment in the three strategies at the population level, we tried to gain further insights into how individual reactions to income inequality evolve over time. In each study, we fitted latent class linear mixed models (LCLMM)^47^ to classify low-income individuals into distinct behavioral types based on the evolution of their investment in the three strategies (see Supporting Information S1 and Tables S6 and S7 for detailed analysis and results). The LCLMM tries to explain between-subject heterogeneity in growth on an outcome by assuming that the population is divided into a finite number of latent subpopulations. Each latent group of participants is characterized by a different growth trajectory (i.e., behavioral type) which is modelled by a class-specific linear mixed model.

### Results

**Figure 4:**
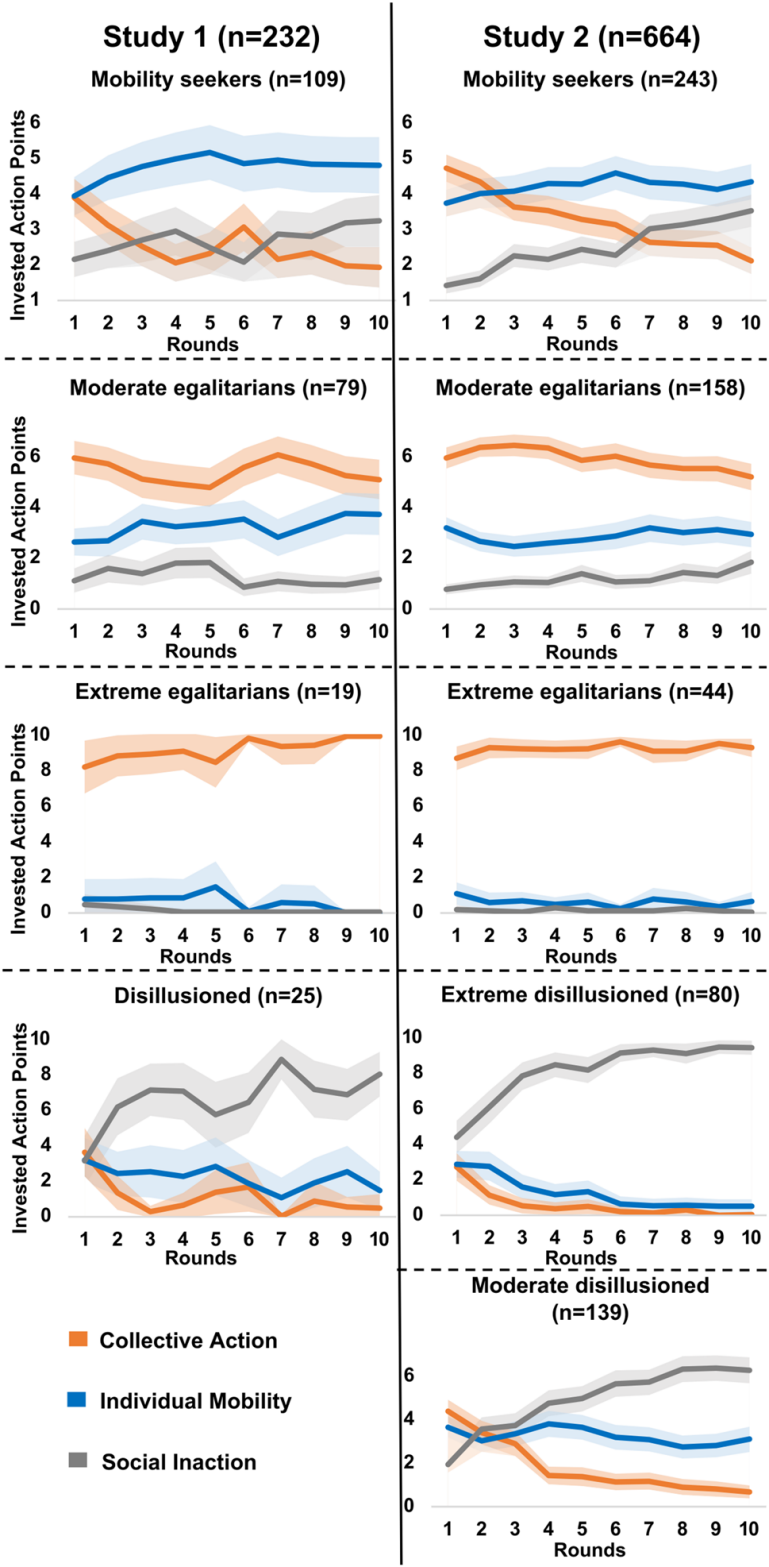
Behavioral types across the two studies. In both Study 1 (left) and Study 2 (right), low-income individuals were classified into three generic behavioral types. Bands around the averages in (**A**) and (**B**) indicate the 95% confidence interval of the mean. “Mobility seekers” persistently pursued an individual mobility strategy and gradually disengaged from collective action. Moderate and extreme “egalitarians” predominantly engaged in collective action. The “disillusioned” gradually disengaged from both risky strategies (mostly redistribution) and opted for social inaction. In Study 2, the “disillusioned’ type was represented more prominently and was divided into a moderate and an extreme version.

The analyses revealed four behavioral types in Study 1 and five behavioral types in Study 2 (Fig. 4). In both studies, we identified three generic behavioral types. The most populated behavioral type was the “mobility seekers” (47% of the sample in Study 1 and 37% of the sample in Study 2) who predominantly strived for individual mobility throughout the ten rounds by consistently investing 37–50% of their APs in this strategy. These individuals gradually shifted away from the redistribution strategy to the two individualistic strategies. The second generic type was the “egalitarians” (42% of the sample in Study 1 and 31% of the sample in Study 2) who persistently engaged in collective action throughout the ten rounds. In both studies, we identified a moderate and an extreme version of “egalitarians”. The moderate “egalitarians” (34% of the sample in Study 1 and 24% of the sample in Study 2) invested 50–63% of their APs in collective action, 25–37% in individual mobility, and only 9–19% in social inaction across the ten rounds. The extreme “egalitarians” (8% of the sample in Study 1 and 7% of the sample in Study 2) overwhelmingly engaged in collective action by investing 80–100% of their resources in this strategy throughout the ten rounds. The third generic type, the “disillusioned” (11% of the sample in Study 1 and 33% of the sample in Study 2), gradually disengaged from both risky strategies (mostly from redistribution) and opted for the risk-free and low-profit social inaction strategy. In Study 2, two versions of “disillusioned” individuals were identified, a moderate (21% of the sample) and an extreme version (12% of the sample). Their main differences lie in the starting point and the final degree of investment in social inaction (Fig. 4). The high prevalence of “disillusioned” individuals in Study 2 corroborates the view that explicit feedback about impermeable group boundaries renders social inaction an attractive alternative strategy for a considerable segment of low-income individuals despite being a low-profit strategy.

**Figure 5:**
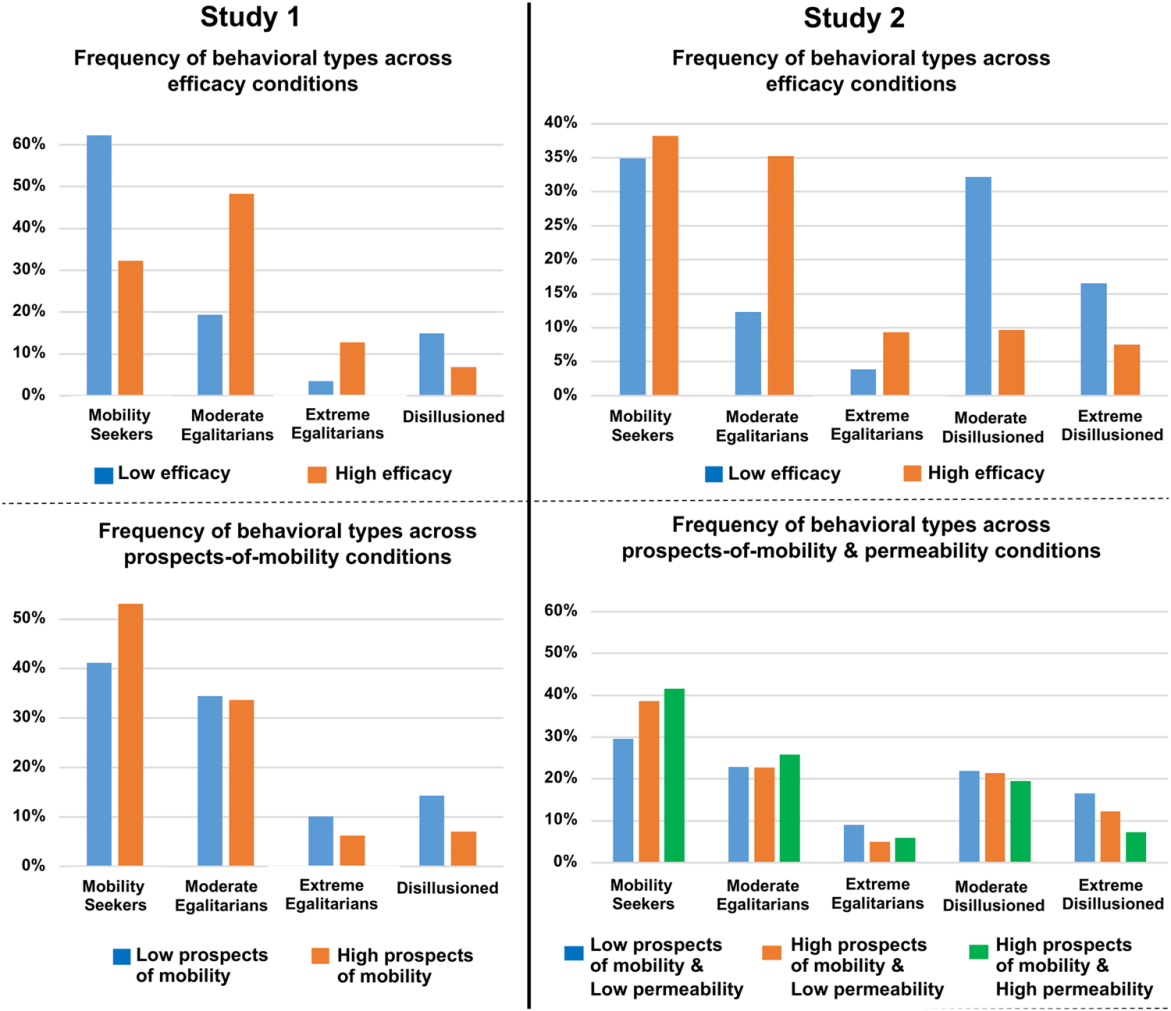
Frequency of each behavioral type across efficacy (upper part), prospects of mobility (lower part), and group permeability (lower part) conditions in both Study 1 (left) and Study 2 (right).

In both Study 1 (χ^2^(3, n = 664) = 35.1, p < 0.001) and Study 2 (χ^2^(4, n = 664) = 96.1, *p* < 0.001), the prevalence of the behavioral types differed significantly across high and low efficacy conditions (Fig. 5 and Tables S8 and S9). As expected, low efficacy decreased the frequency of both moderate and extreme “egalitarians”, while it increased the number of “disillusioned” (in both studies) and “mobility seekers” (only in Study 1). In contrast, exposure to high (vs low) prospects of mobility (in both studies) and high (vs low) group permeability (in Study 2) did not influence the frequency of the behavioral types.

## Discussion

Our work examined how two relevant sociostructural characteristics and perceptions thereof affect disadvantaged individuals’ willingness to collectively redress economic inequality in the presence of two competing individualistic strategies. We showed that low political efficacy of disadvantaged individuals, but not exposure to high prospects of mobility or even high group permeability, undermines public demand for income redistribution. Importantly, the observed differences between the efficacy-based and the mobility-based explanations of tolerance to inequality are not only statistically significant (see comparison of effect sizes in Supporting Information S1 and Tables S2 and S3) but also quantitatively relevant and persistent over time.

Exposure to high prospects of mobility, even when combined with highly permeable income group boundaries, did not undermine collective action against inequality as low-income individuals predominantly shifted from one individualistic strategy (social inaction) to the other (individual mobility). Τhis finding contradicts the prospects of mobility hypothesis^6^ but resonates with prior evidence that permeability of group boundaries and perceptions thereof mainly affect the willingness to engage in individual rather than collective strategies^33^.

One plausible explanation for the lack of mobility-driven effects on collective demand for redistribution is that disadvantaged individuals strive to accomplish two different goals: (1) increase their low personal income and (2) reduce high and unfair intergroup inequality. When income group boundaries are (perceived as) permeable, individual mobility offers an attractive solution to the problem of low personal income without necessarily affecting motivations to reduce high and unfair inequality. Based on this view, previous economic experiments that imposed a negative interdependence between income redistribution and individual mobility^40–42^, were not able to distinguish the two aforementioned goals. Unlike these attempts, we introduced a third risk-free social inaction strategy, which allowed both motivations to be expressed. Nevertheless, the absence of mobility-related effects on collective action may be specific to contexts where inequality is overwhelmingly perceived as unfair. In our setting, this strong sense of unfairness probably contributed to the persistent presence of “egalitarians” across all prospects of mobility & group permeability conditions.

Nonetheless, exposure to high prospects of mobility increased the popularity of individual mobility even in the absence of upward mobility experiences. We initially hypothesized that an early experience of upward mobility (low group permeability in Study 2) combined with exposure to high prospects of mobility would lead to persistent engagement in individual mobility. However, this context failed to produce a long-term prospects-of-mobility effect on individual mobility. Instead, explicit feedback about impermeable group boundaries undermined the longevity of this prospects-of-mobility effect.

This finding suggests that when the absence of upward mobility can be attributed to either impermeable group boundaries or insufficient personal investment (ambiguous feedback of Study 1), exposure to high prospects of mobility exerts a sustained effect on investment in individual mobility and group permeability perceptions. In contrast, when the absence of upward mobility is solely attributed to impermeable group boundaries (explicit feedback of Study 2 after the first round), exposure to high prospects of mobility has a short-lived effect on individual mobility as disadvantaged individuals gradually shift away from individual mobility and adjust downwards their permeability perceptions. The explicitly negative feedback may also explain the substantially higher prevalence of “disillusioned” individuals in this study. However, when group permeability was actually high, the popularity of individual mobility and group permeability perceptions increased over time. Taken together, these results suggest that people incorporate information about actual group permeability, when these are available, and adjust their beliefs and behavior accordingly^9,20^. At the same time, these findings highlight the crucial role of ambiguity regarding group permeability in explaining the persistent optimism about prospects of mobility and preference for individual rather than collective action observed in token systems^16^.

Consistent with the gradual erosion of the public good^44^, collective demand for redistribution started off as the most popular strategy across all conditions, but it gradually declined when disadvantaged individuals lacked political efficacy. This trend probably reflects the presence of conditional cooperators who stopped contributing after failed attempts to collectively reduce inequality (see Supporting Information S1 and Tables S12 and S13 for feedback effects on collective action). Despite the declining collective action, the income gap was persistently deemed unfair and even more so in the low efficacy condition. This discrepancy between beliefs and behavior disproves a system-justifying account whereby powerless individuals legitimize the inequality they tolerate^29^. Instead, it is consistent with evidence that powerless and low-status individuals are more likely to recognize the unfairness of the existing economic system^30,31,48^. A question that remains unaddressed and perhaps an avenue for future research is whether prolonged lack of political efficacy could shape people’s ideology by rendering egalitarianism a utopian ideal rather than a cognitive alternative to a highly unequal system.

Our work builds upon a recent series of public good experiments where individuals could solve shared problems collectively or individually^49,50^. In these studies, people displayed a strong tendency to opt for the individual solution, which resonates with our finding that “mobility seekers” was the most populated behavioral type. These studies also showed that as the collective solution becomes more efficient, people engage more in collective action and vote to abolish individual solutions. Similarly, we found that when disadvantaged individuals have the means to collectively reduce inequality, public demand for redistribution rises. This shift is also reflected at the individual level, as there were three times more “egalitarians” and half as many “disillusioned” individuals when political efficacy was high. In this respect, our results contradict classic instrumental accounts according to which people free-ride when a collective goal becomes feasible without personal contribution^39^. Instead, our results corroborate evidence from the social identity tradition showing that group efficacy beliefs are important predictors of collective action against inequalities^8,32,38^. In fact, the present work goes beyond the existing social identity models of collective responses to inequality by testing some of their assumptions using incentivized behavioral measures rather than group efficacy and group permeability beliefs.

The robust efficacy effect on collective action has profound political implications in light of the changes that characterize many countries in the last three decades. Growing economic inequality is concurrent with a general crisis of democratic institutions, which has resulted in growing disparities between high and low socioeconomic status individuals in political participation^21–26^. To date, the low political efficacy of disadvantaged individuals has been mostly considered as a consequence of economic inequality^27,51–53^. The present work reverses this argument in an attempt to understand how political inequality contributes to the consolidation and perpetuation of economic inequality. The potential self-reinforcing link between economic and political inequality^25^ provides a plausible explanation as to why low public demand for the growing wealth and income gap is more prevalent in highly unequal countries^3^. Consistent with this view, median voter models that take into account the wealth bias in political participation outperform the standard model^5^ in predicting the dynamics of income redistribution^54^.

One important limitation of the present findings is that political efficacy was operationalized as a structural manipulation, while exposure to different mobility prospects is an ideological manipulation given that actual group permeability was the same across conditions. As explained in the introduction, we opted for these two manipulations to increase ecological validity. Nonetheless, the different nature of the two manipulations (i.e., structural vs ideological) may account for the stronger efficacy effects on collective action. To counteract this argument, in Study 2, we manipulated actual group permeability. Interestingly, even this structural manipulation had no effect on collective action.

To conclude, the present work highlights the lack of political efficacy as a plausible explanation of the disadvantaged individuals’ tolerance to growing inequality and casts doubt on the prominent mobility-based explanations especially in contexts where social inaction is an alternative strategy to costly collective action and individual mobility. These results call for more attention on how broader political factors affect motivations of disadvantaged individuals to address economic inequality.

### Supplementary Information

Supplementary Information.

## Data Availability

The data are available via the Open Science Framework at https://osf.io/dqne7.

## References

[1] Alvaredo F, Chancel L, Piketty T, Saez E, Zucman G (2017). Global inequality dynamics: New findings from WID. world. Am. Econ. Rev..

[2] Winkler H (2019). The effect of income inequality on political polarization: Evidence from European regions, 2002–2014. Econ. Polit. Oxford.

[3] Mijs JJB (2021). The paradox of inequality: Income inequality and belief in meritocracy go hand in hand. Socio-Econ. Rev..

[4] Piff PK, Kraus MW, Keltner D (2018). Unpacking the inequality paradox: The psychological roots of inequality and social class. Adv. Exp. Soc. Psychol..

[5] Meltzer AH, Richard SF (1981). A rational theory of the size of government. J. Polit. Econ..

[6] Benabou R, Ok EA (2001). Social mobility and the demand for redistribution: The POUM hypothesis. Q. J. Econ.

[7] Manza J, Brooks C (2021). Mobility optimism in an age of rising inequality. Sociol. Q..

[8] Jetten J (2021). Consequences of economic inequality for the social and political vitality of society: A social identity analysis. Polit. Psychol..

[9] Alesina A, Stantcheva S, Teso E (2018). Intergenerational mobility and preferences for redistribution. Am. Econ. Rev..

[10] Garcia-Sanchez E, Osborne D, Willis GB, Rodriguez-Bailon R (2020). Attitudes towards redistribution and the interplay between perceptions and beliefs about inequality. Br. J. Soc. Psychol..

[11] Lameris MD, Garretsen H, Jong-A-Pin R (2020). Political ideology and the intragenerational prospect of upward mobility. Eur. J. Polit. Econ..

[12] Shariff AF, Wiwad D, Aknin LB (2016). Income mobility breeds tolerance for income inequality: Cross-national and experimental evidence. Perspect. Psychol. Sci..

[13] Corak M (2020). Intergenerational mobility: What do we care about? What should we care about?. Aust. Econ. Rev..

[14] OECD. *Broken Social Elevator?: How to Promote Social Mobility*. (Organization for Economic, 2018).

[15] Grisold A, Theine H (2017). How come we know? The media coverage of economic inequality. Int. J. Commun-Us.

[16] Danaher K, Branscombe NR (2010). Maintaining the system with tokenism: Bolstering individual mobility beliefs and identification with a discriminatory organization. Br. J. Soc. Psychol..

[17] Goudarzi S, Pliskin R, Jost JT, Knowles ED (2020). Economic system justification predicts muted emotional responses to inequality. Nat. Commun..

[18] Jost JT (2019). A quarter century of system justification theory: Questions, answers, criticisms, and societal applications. Br. J. Soc. Psychol..

[19] Davidai S, Gilovich T (2015). Building a more mobile America-One income quintile at a time. Perspect. Psychol. Sci..

[20] Weber, N. Experience and perception of social mobility: A cross-country test of the self-serving bias. (LIS Working Paper Series, 2020).

[21] Gilens M, Page BI (2014). Testing theories of American politics: Elites, interest groups, and average citizens. Perspect. Polit..

[22] Hufe P, Peichl A, Weishaar D (2022). Lower and upper bound estimates of inequality of opportunity for emerging economies. Soc. Choice Welfare.

[23] Bartels, L. M. in *Unequal Democracy* (Princeton University Press, 2016).

[24] Solt F (2008). Economic inequality and democratic political engagement. Am. J. Polit. Sci..

[25] Stiglitz, J. E. The price of inequality: How today's divided society endangers our future (2015).

[26] Amna E, Ekman J (2014). Standby citizens: Diverse faces of political passivity. Eur. Polit. Sci. Rev..

[27] Loveless M (2013). The deterioration of democratic political culture: Consequences of the perception of inequality. Soc. Just. Res..

[28] Benabou R, Tirole J (2006). Belief in a just world and redistributive politics. Q. J. Econ..

[29] van der Toorn J (2015). A sense of powerlessness fosters system justification: Implications for the legitimation of authority, hierarchy, and government. Polit. Psychol..

[30] Brandt MJ (2020). Subjective status and perceived legitimacy across countries. Eur. J. Soc. Psychol..

[31] Buchel O, Luijkx R, Achterberg P (2021). Objective and subjective socioeconomic status as sources of status-legitimacy effect and legitimation of income inequality. Polit. Psychol..

[32] van Zomeren M, Postmes T, Spears R (2008). Toward an integrative social identity model of collective action: A quantitative research synthesis of three socio-psychological perspectives. Psychol. Bull..

[33] Ellemers N, Vanknippenberg A, Wilke H (1990). The influence of permeability of group boundaries and stability of group status on strategies of individual mobility and social-change. Br. J. Soc. Psychol..

[34] Hersby MD, Ryan MK, Jetten J (2009). Getting together to get ahead: The impact of social structure on women's networking. Br. J. Manage.

[35] Mummendey A, Kessler T, Klink A, Mielke R (1999). Strategies to cope with negative social identity: Predictions by social identity theory and relative deprivation theory. J. Pers. Soc. Psychol..

[36] Wright SC, Taylor DM, Moghaddam FM (1990). Responding to membership in a disadvantaged group—From acceptance to collective protest. J. Pers. Soc. Psychol..

[37] van Zomeren M (2016). Building a tower of babel? Integrating core motivations and features of social structure into the political psychology of political action. Polit. Psychol..

[38] van Zomeren M, Saguy T, Schellhaas FMH (2013). Believing in "making a difference" to collective efforts: Participative efficacy beliefs as a unique predictor of collective action. Group Process Interg..

[39] Olson, M. *The logic of collective action*. Vol. 124 (Harvard University Press, 2009).

[40] Agranov M, Palfrey TR (2020). The effects of income mobility and tax persistence on income redistribution and inequality. Eur. Econ. Rev..

[41] Checchi, D. & Filippin, A. in *Inequality, welfare and income distribution: Experimental approaches* (Emerald Group Publishing Limited, 2004).

[42] Jiménez, N., Molis, E. & Solano-García, A. Why do the poor vote for low tax rates? A (real-effort task) experiment on income redistribution. (2019).

[43] Wright, S. C. Restricted intergroup boundaries—Tokenism, ambiguity, and the tolerance of injustice. *Psychol. Legit.* 223–254 (2001).

[44] Ledyard, J. O. Is there a problem with public goods provision. *Handb. Exp. Econ.* 111–194 (1995).

[45] Chen DL, Schonger M, Wickens C (2016). oTree-An open-source platform for laboratory, online, and field experiments. J. Behav. Exp. Financ..

[46] Abeler J, Falk A, Goette L, Huffman D (2011). Reference points and effort provision. Am. Econ. Rev..

[47] Wardenaar, K. Latent class growth analysis and growth mixture modeling using R: A tutorial for two R-packages and a comparison with Mplus. (2020).

[48] Lois G, Riedl A (2022). Interplay between different forms of power and meritocratic considerations shapes fairness perceptions. Sci. Rep..

[49] Gross J, Bohm R (2020). Voluntary restrictions on self-reliance increase cooperation and mitigate wealth inequality. Proc. Natl. Acad. Sci. U.S.A..

[50] Gross J, De Dreu CKW (2019). Individual solutions to shared problems create a modern tragedy of the commons. Sci. Adv..

[51] Houle C (2018). Does economic inequality breed political inequality?. Democratization.

[52] Lee D, Chang CY, Hur H (2021). Political consequences of income inequality: Assessing the relationship between perceived distributive fairness and political efficacy in Asia. Soc. Justice Res..

[53] Schafer A, Schwander H (2019). 'Don't play if you can't win': Does economic inequality undermine political equality?. Eur. Polit. Sci. Rev..

[54] Brendler, P. Income inequality and political inequality in the US. (2014).

