# Supplementary material for: Explaining inequality tolerance in the lab: effects of political efficacy and prospects of mobility on collective demand for redistribution
Source: Sci Rep. 2023 Sep 23;13:15872. doi: 10.1038/s41598-023-42715-9 (PMC10517979; doi:10.1038/s41598-023-42715-9)
Supplement: Supplementary file 1 — Supplementary Information. [file 41598_2023_42715_MOESM1_ESM.docx]

**Supporting Information for**

**Explaining inequality tolerance in the lab: Effects of political efficacy and prospects of mobility on collective demand for redistribution**

Giannis Lois^ab^, Katerina Petkanopoulou^bc^

^a^Department of Microeconomics and Public Economics, School of Business and Economics, Maastricht University, 6200 MD Maastricht, The Netherlands

^b^Department of Psychology, Faculty of Social Sciences, University of Crete, Greece

^c^Department of Psychology, Panteion University of Athens, Greece

**This PDF file includes:**

Supporting text

Tables S1 to S14

SI References

Table of contents

The supporting information is divided into three parts.

In the first part (I Instructions), we provide details on the experimental procedure including instructions and information provided to participants in each screen.

In the second part (II Preregistered hypotheses), we report and test all preregistered hypotheses and we present the results.

In the third part (II Extended results Study 1 and Study 2), we provide detailed information on the analyses underlying the results reported in the main manuscript.

In the fourth part (III Additional and exploratory analyses), we report additional analyses that are not reported in the main manuscript and were not the main focus of the study, such as analyses including covariates and effects tested for exploratory purposes.

[I Instructions 3](#_Toc144633681)

[II Preregistered hypotheses 13](#_Toc144633682)

[Study 1 13](#_Toc144633683)

[Study 2 (first preregistration) 15](#_Toc144633684)

[Study 2 (second preregistration) 16](#_Toc144633685)

[III Extended results Study 1 and Study 2 18](#_Toc144633686)

[Demographics 18](#_Toc144633687)

[Comparing effect sizes 19](#_Toc144633688)

[Effects on beliefs 20](#_Toc144633689)

[Growth mixture model analysis 23](#_Toc144633690)

[IV Additional and exploratory analyses 27](#_Toc144633691)

[Robustness test: Testing the effect of covariates 27](#_Toc144633692)

[Feedback effects on collective action 29](#_Toc144633693)

[Experiencing vs witnessing upward mobility 31](#_Toc144633694)

[Figure S1 33](#_Toc144633695)

[SI References 35](#_Toc144633696)

# I Instructions

**Screen 1**

**Introduction**

Welcome to this study which will last approximately **25 minutes**.

This study has two phases: (1) an **income production phase** and (2) a **decision phase.**

With your participation in this study, you can earn money, which will be paid out in three parts:

1. **Participation Fee**: You will receive a **fixed participation fee of 2.15£**.
2. **Comprehension Bonus:** It is very important that you understand well the instructions. Before the main part of the study, you will answer some comprehension questions. For **each comprehension question** you answer correctly **on your first attempt**, you will earn **£0.10.**
3. **Income:** During the decision phase, the size of the income you have produced can change based on your decisions and the decisions of other participants. The final income can range from **1£ to 5£**.

**Screen 2**

This study involves **real-time interaction with other participants**.

Thus, it is important that **you complete the entire study.**

**To avoid waiting for the others for too long,** there is a maximum amount of time you and the other participants can spend in each Screen.

**Attention:** If you fail to submit your response within the given time in **two successive Screens**, **you will be excluded from the study.**

**Screen 3**

**Income production phase**

First, you will perform a **Counting Zeros task** to produce your income.

During this task, you have to **count how many zeros (0) are contained in 5x5 tables of numbers** and write the number of zeros in a box next to each table (see example below).

You have **2 minutes** at your disposal to solve as many tables as possible by counting the zeros correctly.

**To produce your income, the number of tables you solve should exceed a threshold that is unknown to you.**

Since the threshold is unknown, **you need to solve as many tables as possible within 2 minutes** to increase **your chances** of producing your income.

Below, you can see an example of the task with three solved tables:


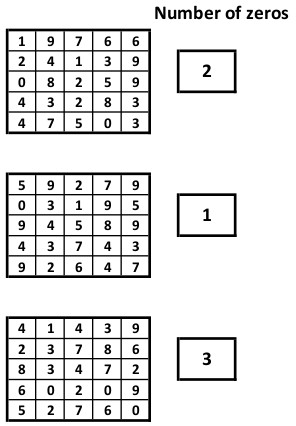


**Screen 4**

**Two groups and two different incomes**

Like **you, seven other participants** exceeded the unknown threshold and produced their income.

You and these seven other participants will be **randomly assigned** to either a **high-income** or a **low-income** group:

1. **Four participants** will be **randomly assigned** to the **high-income group** and their income will be **5£.**
2. The other **four participants** will be **randomly assigned** to the **low-income group** and their income will be **1£.**

Now, **the computer will randomly assign** you and the other seven participants to either the **low-income** or the **high-income group.**

**Screen 5**

**You** were **randomly assigned** to the **low-income group.**

**Low-income** members receive **£1** as income.

**High-income** members receive **£5** as income.

**Screen 6**

**Action Points**

**Low-income members (including you)** are endowed with **10 Action Points** which they can use **to increase their income** in the following three ways:

1. The four low-income members can contribute their Action Points to a **public pool** to achieve **redistribution of income from the high-income group to the low-income group (i.e., reduce income differences between groups).**
2. Each low-income member can allocate his/her Action Points to a **personal account** to increase his/her chances of **moving to the high-income group.**
3. Each low-income member can **exchange his/her Action Points for money.**

**Low-income** **members** can allocate their **10 Action Points any way they want.**

**Here is a hypothetical example of how a low-income member used his/her 10 Action Points**

**Screen 7**

**Twelve Independent Rounds**

Low-income members are given twelve independent chances (twelve rounds) to increase their income.

**Each of the twelve rounds is independent from other rounds or in other words, what happens in one round does not affect the next round:**

1. If a low-income member moves to the high-income group in a certain round, in the next round he/she will again start as a low-income member.

2. Even when there is income redistribution in one round, in the next round the initial income of the low-income group is £1 and the initial income of the high-income group is £5.

At the end of each round, low-income members will receive feedback about income redistribution in this round and who moved to the high-income group in this round.

Each participant will receive as bonus payment the final income of his/her group in one randomly selected round plus the money he/she received from exchanging his/her Action Points in the selected round.

**Screen 8**

**First way of using Action Points:**

**Redistributing income between the two groups**

If the four low-income members contribute, in total, from 12 to 23 (from 28 to 39) Action Points to the public pool, the income of the low-income group increases from £1 to £2 and the income of the high-income group decreases from £5 to £4.

If the four low-income members contribute, in total, 24 or more (40) Action Points to the public pool, the income of the low-income group increases from £1 to £3 and the income of the high-income group decreases from £5 to £3.

The table below depicts how Action Points contributed to the public pool affect the income of both groups:

**Low efficacy Condition:**

| **Total Action Points contributed to the public pool** | **Income of low-income group** | **Income of high-income group** |
| --- | --- | --- |
| **0 – 11** | **£1** | **£5** |
| **12 - 23** | **£2** | **£4** |
| **24 – 40*** | **£3** | **£3** |

*Each low-income member is endowed with 10 Action Points and thus in total the four low-income members have 40 Action Points

**High efficacy Condition:**

| **Total Action Points contributed to the public pool** | **Income of low-income group** | **Income of high-income group** |
| --- | --- | --- |
| **0 – 27** | **£1** | **£5** |
| **28 - 39** | **£2** | **£4** |
| **40*** | **£3** | **£3** |

*Each low-income member is endowed with 10 Action Points and thus in total the four low-income members have 40 Action Points

**Screen 9**

**Second way of using Action Points:**

**Moving to the high-income group**

Some of the twelve rounds may be **moving rounds**. In a moving round, the **two, out of the four, low-income members** who allocated the **most Action Points to their personal account** will move to the high-income group.

At the end of the experiment, all low-income members, including you, are asked how likely it is that a randomly selected round is a moving round.

According to some low-income members of previous sessions**, the probability that a randomly selected round is a moving round is around 10% (50%).**


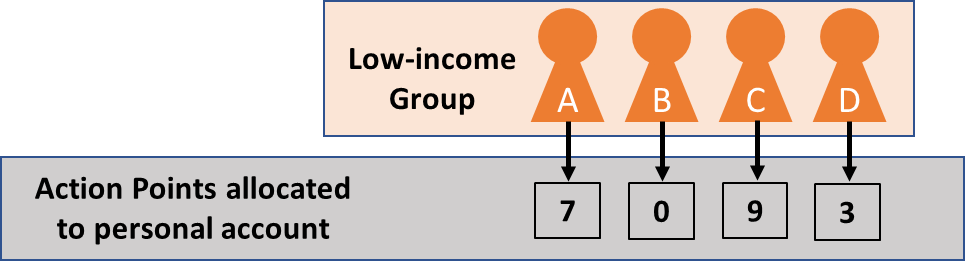


If this hypothetical round is a moving round, then players A and C move to the high-income group.


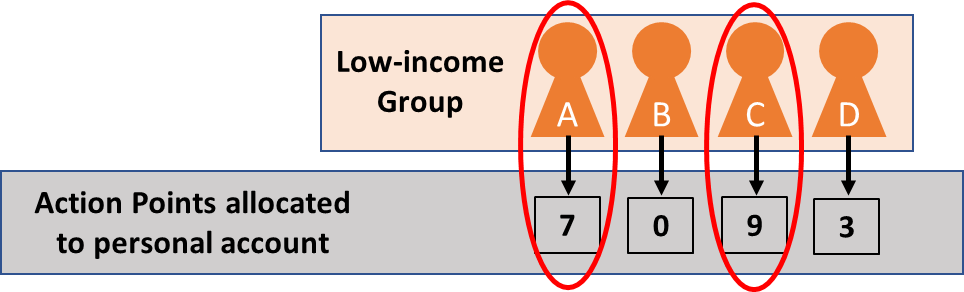


**Screen 10**

**Third way of using Action Points:**

**Exchange Action Points for money**

Low-income members can exchange their Action Points for money.

**For each Action Point** low-income members exchange, they receive **£0.05.**

**Example 1:** A low-income member who exchanges his/her **2 Action Points** for money will receive an additional income of **£0.10.**

**Example 2:** A low-income member who exchanges his/her **10 (all) Action Points** for money will receive an additional income of **£0.50.**

**Attention:** Any money that result from exchanging Action Points will be **added to the final income of this round**.

**Screen 11**

**Comprehension Question 1**

You have one attempt to answer the following question correctly and earn a **bonus of £0.10.**

If your answer is **wrong,** you will **receive feedback** about the correct answer.

**Question:** A low-income member moves to the high-income group in the **fourth round.**

In which income group will he/she be a member of **at the start of the fifth (next) round?**

1. **High-income group**
2. **Low-income group**
3. **None of the above**

**Screen 12**

**Comprehension Question 2**

You have one attempt to answer the following question correctly and earn a **bonus of £0.10.**

If your answer is **wrong,** you will **receive feedback** about the correct answer.

**Question:** In the fourth round, **income redistribution** takes place so that the final incomes of both low-income group and high-income group are **£3.**

Please choose the **initial income** of the low-income group **in the fifth (next) round:**

1. **£1**
2. **£2**
3. **£3**

**Screen 13**

**Comprehension Question 3**

You have one attempt to answer the following question correctly and earn a **bonus of £0.10.**

If your answer is **wrong,** you will **receive feedback** about the correct answer.

**Question:** In the example below, what is the **final income** of the low-income group?

1. **£5**
2. **£2**
3. **£3**
4. **£1**


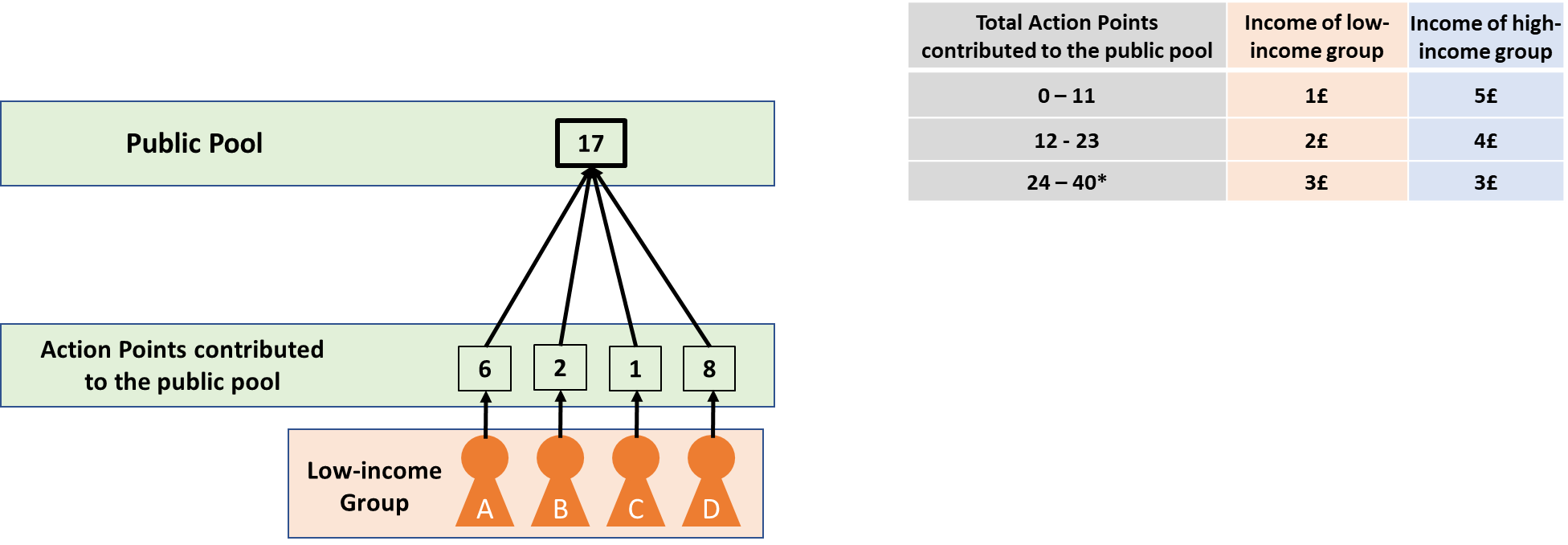


**Screen 14**

**Comprehension Question 4**

You have one attempt to answer the following question correctly and earn a **bonus of £0.10.**

If your answer is **wrong,** you will **receive feedback** about the correct answer.

**Question:** Let’s assume that the hypothetical round below is a **moving round**.

Which two low-income members will move to the high-income group in this hypothetical round?

1. **A** and **B**
2. **C** and **D**
3. **B** and **D**
4. **A** and **C**


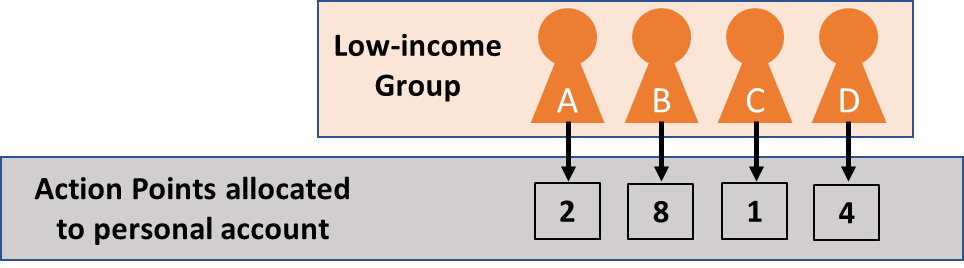


**Screen 15**

**Action Points Rounds**

Now, you and the other three low-income members will play **twelve independent rounds** in which you can use your Action Points to **increase your income**.

This part of the study will start when **all four low-income members have read the instructions.**

**Please wait …**

**This may take a few minutes. Thank you for your patience.**

**Screen 16**

**New/First round starts**.

Please decide **how to use your 10 Action Points** in this round.

**First way:** You and the other three low-income members can contribute your Action Points to the **public pool** to achieve **income redistribution from the high-income group to the low-income group** in the following way:


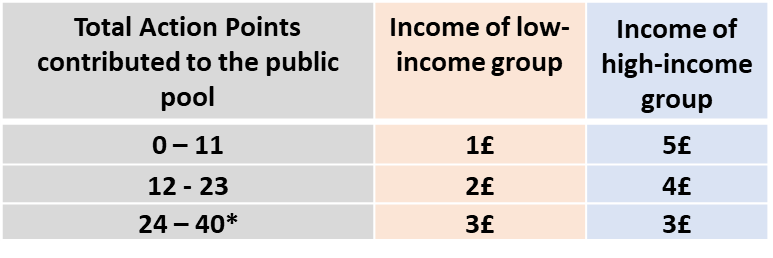


**Second way:** You can allocate your Action Points to **your personal account**.

According to some low-income members of previous sessions, **the** **probability that a randomly selected round is a moving round is around 10% (50%).**

If this is a moving round, the **two low-income members who allocate the most Action Points to their personal account will move to the high-income group.**

**Third way:** You exchange your Action Points for money. **For each Action Point you exchange, you earn 0.05£.**

- **Action Points you want to contribute to the public pool: ____**
- **Action Points you want to allocate to your personal account: ____**
- **Action Points you want to exchange for money: _____**

**Screen 17 (Redistribution outcome: No inequality reduction)**

**Feedback: Income redistribution**

**You** contributed **X** Action Points to the **public pool**.

The **four low-income members, including you,** contributed to the public pool in total **less than 12 (28) Action Points.**

Therefore, the final income of the two groups are:

**Low-income group = £1**

**High-income group = £5**

**Screen 17 (Redistribution outcome: Reduced inequality)**

**Feedback: Income redistribution**

**You** contributed **X** Action Points to the **public pool**.

The **four low-income members, including you,** contributed to the public pool in total **between 12 and 23 (28 and 39) Action Points.**

Therefore, the final income of the two groups are:

**Low-income group = £2**

**High-income group = £4**

**Screen 17 (Redistribution outcome: Absolute equality)**

**Feedback: Income redistribution**

**You** contributed **X** Action Points to the **public pool**.

The **four low-income members, including you,** contributed to the public pool in total **more than 23 (40) Action Points.**

Therefore, the final income of the two groups are:

**Low-income group = £3**

**High-income group = £3**

**Screen 18 (Study 1)**

**Negative Feedback: Moving to the high-income group**

You allocated 5 Action Points to your personal account.

In this round, you do not move to the high-income group.

There are two possible reasons for this:

1. Either because **this round is not a moving round.**

Remember that according to some low-income members of previous sessions, the probability that a randomly selected round is a moving round is around 10%(50%).

1. Or this is a moving round but **two other low-income members allocated more Action Points** than you to their personal account.

**Screen 18 (Study 2)**

**Negative Feedback: Moving to the high-income group**

You allocated 5 Action Points to your personal account.

**This round is not a moving round.**

No low-income member moves to the high-income group and thus your income is £1.

According to low-income members of previous sessions, the probability that a randomly selected round is a moving round is around **10%(50%)**

**Positive Feedback: Moving to the high-income group**

You allocated 5 Action Points to your personal account.

**Τhis round is a moving round.**

You and another low-income member (do not) move to the high-income group because the two of you (the two other low-income members) allocated more points than the other low-income members (you) in your (their) personal accounts.

Since you (did not) move(d) to the high-income group your income is £5 (£1)

According to low-income members of previous sessions, the probability that a randomly selected round is a moving round is around **10% (50%).**

**Screen 19**

**Feedback: Exchange for money**

You exchanged 5 Action Points for money and thus you earned an additional income of £0.25.

**Screen 20 (only at the end of first and tenth round)**

How **fair or unfair** do you find the **income gap between low-income and high-income group**?


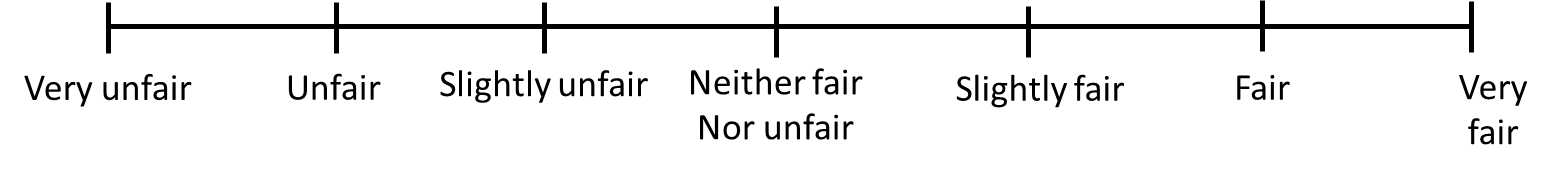


**Screen 21 (only at the end of first and tenth round)**

In each round, how **likely** it is for a low-income member **to move to the high-income group** through the points they allocate to their personal account?

**
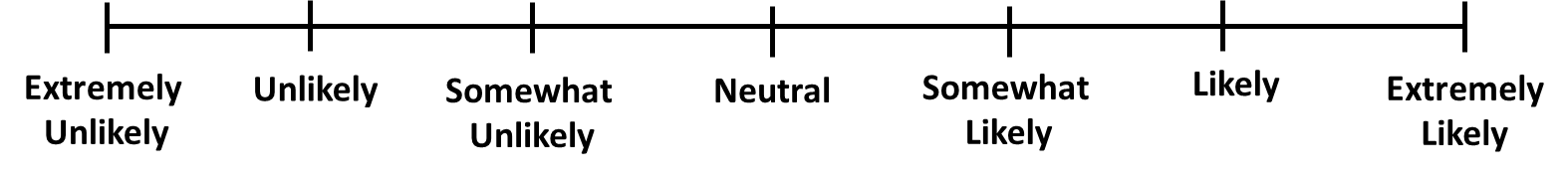
**

**Screen 22 (only at the end of first and tenth round)**

To what extent, low-income members can **collectively achieve a higher income for their group** through their total contributions to the public pool?

**
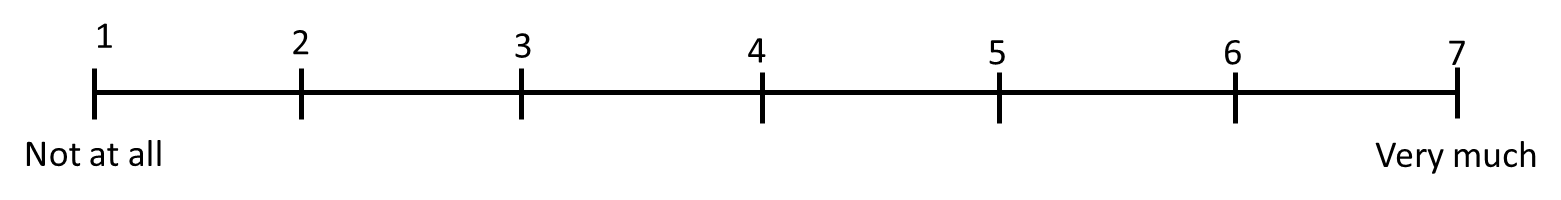
**

**Screen 23 (only at the end of first and tenth round)**

Please estimate the **total amount of Action Points that were contributed to the public pool** by the four low-income members in this round.

**
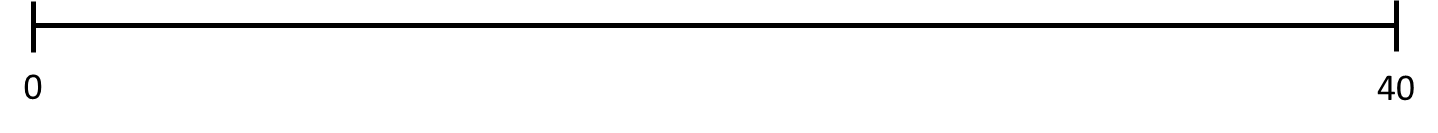
**

**Screen 24 (only at the end of tenth round)**

To what extent do you **identify with other low-income members?**

**
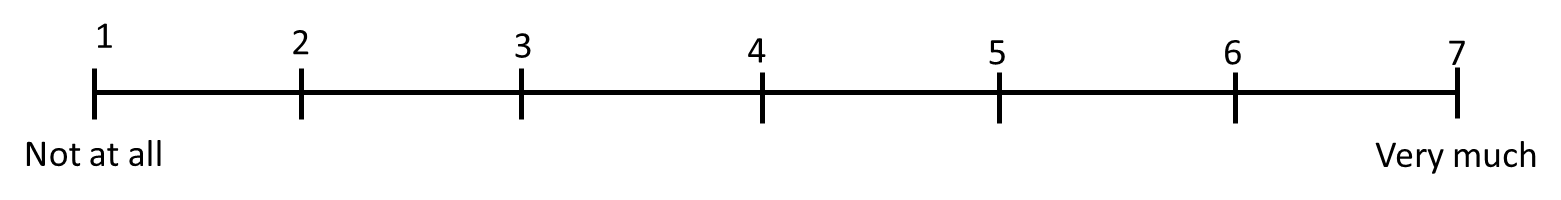
**

**Screen 25 (only at the end of 12^th^ round)**

What is the **probability** that one randomly selected round out of the twelve rounds is **a mobility round**?


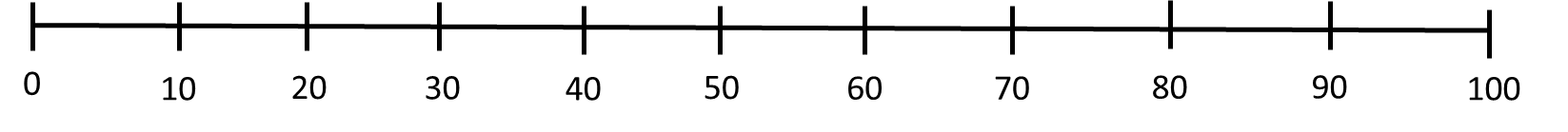


**Screen 27**

To what extent do you **identify with other low-income members?**

**
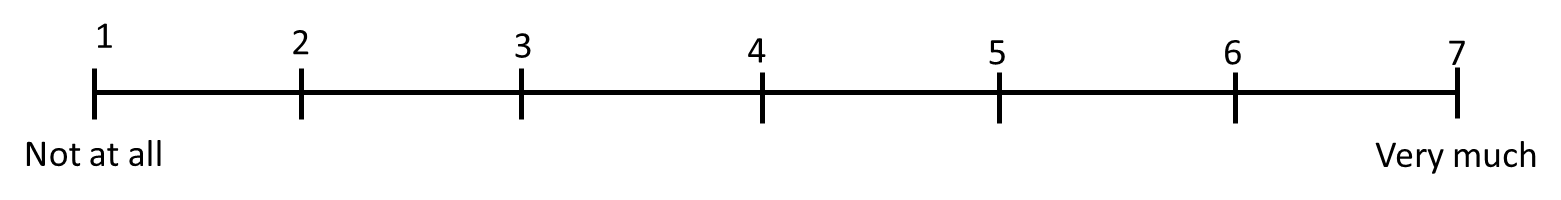
**

**Screen 27**

**Debriefing**

The aim of this study is to investigate how people react in unequal distribution of resources. The existing literature points to two different types of reactions. The collective ones when people try to improve the position of the group as whole and the individual one when people try to improve their own position. In this study we investigate the factors that might affect whether people opt to react either collectively or individually.

This study was indeed an interactive study where you and three other participants made real-time decisions as members of the low-income group. In each round, you received real feedback about the contributions of low-income members to the public pool and the resulting redistribution of income from the high-income to the low-income group.

However, **this study included one aspect of deception.**

You were informed that you were randomly assigned to either a low-income or high-income group. However, the high-income group did not really exist, and **all participants were assigned to the low-income group**. The reason for this deception aspect is that this study focuses on how members of the low-income group react to inter-group inequality.

**Screen 28**

**Informed consent**

After receiving complete information about the purpose of this study, I restate my consent to participate in this study and re-give my permission to use my data for scientific purposes.

(If after being fully informed, you want to revoke your consent you can simply close your browser Screen. In that case, you will still receive the participation fee, but you will not receive the income as bonus payment.)

- **no option (if you revoke your consent, you will only receive the participation fee but no bonus payment)**
- **yes option**

# II Preregistered hypotheses

In both Study 1 and Study 2, some preregistered hypotheses were not presented in the manuscript to increase clarity and focus on the main objectives of the present work. Below, we present all preregistered hypotheses and we test them using the preregistered analysis.

## Study 1

Hypotheses about each condition:

H1. In the low prospects-of-mobility and high efficacy condition, the collective action strategy would be more popular than the other two strategies as collective action is clearly the most efficacious strategy (H1a). This effect would either remain stable over time (H1b) or increase (H1c) as feedback of successful collective action reinforces people’s tendency to opt for this strategy.

H1a is confirmed as collective action is clearly the most popular strategy (main strategy effect: *F*(2, 1668) = 19.56, *p* < .001, η^2^ = .243). H1b is confirmed as collective action remains the most popular strategy throughout the experiment (strategy by time interaction effect: *F*(18, 12104) = 1.25, *p* < .213, η^2^ = .020).

H2. In the high prospects-of-mobility and low efficacy condition, the mobility strategy would be more popular than the other two strategies as it is perceived as the most efficacious strategy (H2a). This effect would either remain stable over time (H2b) or become smaller if people opt away from individual mobility after the repeated failure of this strategy (H2c).

H2a is confirmed as individual mobility is the most popular strategy (except from the two first rounds) (main strategy effect: *F*(2, 1931) = 7.83, *p* < .001, η^2^ = .123). Both H2b and H2c are not confirmed as the popularity of individual mobility steeply increases in the first two rounds and remains high in the remaining eight rounds (strategy by time interaction effect: *F*(18, 12842) = 2.67, *p* < .001, η^2^ = .045).

Effects on collective action:

H3. Compared to the low efficacy treatment, high collective action efficacy would increase collective action (H3a). The efficacy effect on collective action would be more pronounced in the low prospects-of-mobility condition where the alternative strategy of individual mobility can be perceived as less attractive (H3b).

Both H3a and H3b were not confirmed as there was no main effect of efficacy on collective action (*F*(1, 228) = 3.00, *p* = .085, η^2^ = .010) and no significant efficacy by prospects-of-mobility interaction effect (*F*(1, 228) = 0.12, *p* = .728, η^2^ = .001).

H4. If people incorporate feedback about income redistribution into their future behavior, then the efficacy effect on collective action would increase over time as the experienced success (high efficacy condition) or failure (low efficacy condition) of collective action would reinforce the initial efficacy effect (H4a). This efficacy by time interaction effect would be more pronounced in the low prospects-of-mobility condition as the presence of an attractive individual strategy (i.e., high prospects-of-mobility condition) would undermine the main effect of efficacy on collective action (H4b similar to H3b).

H4a (H1 in the manuscript) is confirmed as low (vs high) efficacy gradually reduced collective action (*F*(1, 228) = 35.34, *p* < .001, η^2^ = .130). H4b is not confirmed as there is no significant efficacy by prospects-of-mobility by time interaction effect (*F*(1, 228) = 0.15, *p* = .701, η^2^ = .001)

Effects on individual mobility

H5. Compared to the low prospects-of-mobility treatment, exposure to high prospects-of-mobility would increase engagement in individual mobility (H5a). The prospects-of-mobility effect on individual mobility would be more pronounced in the high efficacy condition (H5b). This prospects-of-mobility by efficacy interaction effect is consistent with the assumption that individual mobility will be popular when the mobility strategy is more or equally attractive to collective action. However, when low prospects-of-mobility is combined with high collective action efficacy then most people will opt for collective action and thus they will opt away from individual mobility.

H5a (H2 in the manuscript) is confirmed as high (vs low) prospects of mobility increased investment in individual mobility (main prospects-of-mobility effect: *F*(1, 228) = 6.93, *p* = .009, η^2^ = .030). H5b is not confirmed as there is no significant prospects-of-mobility by efficacy interaction effect on investment in individual mobility (*F*(1, 228) = 0.12, *p* = .728, η^2^ = .001).

H6. If people opt away from individual mobility after repeated failure of this strategy, then the prospects-of-mobility effect on individual mobility would decrease over time due (H6a). On the other hand, if people ignore the repeated failure to achieve individual mobility, then the prospects-of-mobility effect on individual mobility will remain stable over time (H6b).

H6b is confirmed as there is no significant prospects-of-mobility by time interaction effect on investment in individual mobility (*F*(1, 228) = 0.15, *p* = .694, η^2^ = .001).

Feedback-dependent hypothesis

H7. The efficacy effect on the change of collective action over time (H4) would be mediated by the feedback that participants receive at the end of each round about income redistribution. Feedback that indicates no income redistribution (more likely in the low efficacy treatment) would lead to a negative reinforcement loop of low collective action in future rounds to avoid the costs of others’ free riding on them. On the other hand, feedback that indicates substantial income redistribution (more likely in the high efficacy treatment) would lead to a positive reinforcement loop of high collective action in future rounds.

This hypothesis was not formulated properly as it ignored the fact that collective action in the current round affects both the mediator and the dependent variable thus increasing the chance of observing the opposite mediating effect from the one we hypothesized. The lower collective action is in one round the less likely it is that income redistribution will occur in this round (mediator) but the more likely it is that collective action in the next round will be higher (i.e., positive change in collective action and negative indirect effect). In contrast, the higher collective action is in one round the more likely it is that income redistribution will occur in this round (mediator) but the less likely it is that collective action in the next round will be higher (i.e., negative change in collective action and negative indirect effect).

## Study 2 (first preregistration)

H1. In the low prospects-of-mobility and high efficacy condition, the collective action strategy would be more popular than the other two strategies as collective action is presented as (and actually is) the most efficacious strategy (H1a). This effect would remain stable over time (no increase) as free riding remains attractive when collective action in the group is high (H1b).

H1a is confirmed as collective action strategy is significantly more popular than the other two strategies (main strategy effect: *F*(2, 1705) = 29.17, *p* < .001, η^2^ = .207). H1b is not confirmed as there was a significant strategy by time interaction effect in that investment in collective action gradually declined while investment in social inaction gradually increased (*F*(18, 12632) = 4.42, *p* < .001, η^2^ = .038).

H2. In the high prospects-of-mobility and low efficacy condition, the mobility strategy would be more popular than the other two strategies as it is perceived as the most efficacious strategy (H2a). This effect would remain stable over time despite the repeated cancellation of mobility expectations as people would prefer to hold an optimistic view of future upward mobility rather than update their mobility expectations based on evidence (H2b).

Although there is a main effect of strategy on investment, H2a is not confirmed as individual mobility was not the most popular strategy across time. H2b is not confirmed as investment in individual mobility and collective action gradually declined as participants shifted to social inaction over time.

H3. In the high prospects-of-mobility and high efficacy condition, the social inaction strategy would be less popular than the other two strategies as it is perceived as the least efficacious strategy.

H3 is partially confirmed as social inaction is the least popular strategy in the first two rounds but over time the popularity of this strategy increased while investment in collective action and individual mobility gradually declined.

Effects on collective action:

H4. Compared to the low efficacy treatment, high collective action efficacy would increase collective action (H4a). The efficacy effect on collective action may be more pronounced in the low prospects-of-mobility condition where the alternative strategy of individual mobility can be perceived as less attractive (H4b).

H4a is confirmed as high (vs low) efficacy increased collective action (main efficacy effect: *F*(1, 439) = 13.17, *p* < .001, η^2^ = .030). H4b is not confirmed as there is no significant efficacy by prospects-of-mobility interaction effect on collective action (*F*(1, 439) = 0.12, *p* = .726, η^2^ < .001).

H5. If people incorporate feedback about income redistribution into their future behavior, then the efficacy effect on collective action would increase over time as the experienced success (high efficacy condition) or failure (low efficacy condition) of collective action would reinforce the initial efficacy effect.

H5 (H1 in the manuscript) is confirmed as there is a significant efficacy by time interaction effect on collective action (*F*(1, 439) = 21.81, *p* < .001, η^2^ = .050).

Effects on individual mobility

H6. Compared to the low prospects-of-mobility treatment, exposure to high prospects of mobility would increase engagement in individual mobility.

H6 (H2 in the manuscript) is confirmed as high (vs low prospects of mobility) increased investment in individual mobility (main prospects-of-mobility effect: *F*(1, 439) = 24.57, *p* < .001, η^2^ = .050).

H7. The experience of mobility in the first round may lead to a sustained investment in the mobility strategy in the high prospects-of-mobility treatment but not in the low prospects-of-mobility treatment. Therefore, the prospects-of-mobility effect would increase over time.

H7 (H3b in the manuscript) is not confirmed as there is no significant prospects-of-mobility by time interaction effect on investment in individual mobility (*F*(1, 439) = 0.10, *p* = .745, η^2^ < .001).

H8. Compared to the high efficacy treatment, low collective action efficacy would increase individual mobility as people will switch from a collective action strategy to an individual mobility.

H8 is confirmed as low (vs high) efficacy increased investment in individual mobility (main efficacy effect: *F*(1, 439) = 10.82, *p* < .001, η^2^ = .020).

H9. To the extent that the efficacy effect on collective action increases over time, we would expect a time by efficacy effect on individual mobility as well: low efficacy would lead to a gradual increase of individual mobility compared to high efficacy treatment.

H9 is not confirmed as there is no significant efficacy by time interaction effect on investment in individual mobility (*F*(1, 439) = 3.45, *p* = .064, η^2^ = .008).

## Study 2 (second preregistration)

H1. Compared to the high prospects-of-mobility & low permeability condition, high prospects-of-mobility & high permeability would decrease collective action (H1a). The permeability effect on collective action may be more pronounced in the low efficacy condition where the strategy of individual mobility is clearly more attractive (H1b).

H1a is not confirmed as high (vs low) permeability did not increase collective action (main permeability effect: *F*(1, 437) = 0.24, *p* = .626, η^2^ = .001). H1b is not confirmed as there is no permeability by efficacy interaction effect on collective action (*F*(1, 437) = 0.65, *p* = .421, η^2^ = .001).

H2. As people experience or witness mobility, they are more likely to invest in this strategy and refrain from engaging in collective action. Thus, we expect that the permeability effect on collective action will increase over time (H2a). This time by permeability effect may be more pronounced in the low efficacy condition as the permeability-driven reduction of collective action may be compromised when collective action efficacy is high (H2b).

H2a (H4 in the manuscript) is not confirmed as there is no permeability by time interaction effect on collective action (*F*(1, 437) = 0.07, *p* = .792, η^2^ < .001). H2b is not confirmed as there is no significant permeability by efficacy by time interaction effect on collective action (*F*(1, 437) = 4.85, *p* = .028, η^2^ = .010).

Effects on individual mobility

H3. Compared to the high prospects-of-mobility & low permeability condition, high prospects-of-mobility & high permeability would increase engagement in individual mobility (H3a). The permeability effect on individual mobility may be more pronounced in the low efficacy condition where the strategy of individual mobility is clearly more attractive (H3b).

H3a is not confirmed as there is no significant main effect of permeability on investment in individual mobility (*F*(1, 437) = 1.58, *p* = .209, η^2^ = .004). H3b is not confirmed as there is no significant permeability by efficacy interaction effect on investment in individual mobility (*F*(1, 437) = 2.75, *p* = .098, η^2^ = .006).

H4. As people experience or witness mobility, they are more likely to invest in this strategy. Thus, we expect that the high-mobility effect on collective action will increase over time (H4a). This time by mobility effect may be more pronounced in the low efficacy condition as the mobility-driven increase of mobility may be compromised when collective action efficacy is high (H4b).

H4a (H5 in the manuscript) was confirmed as high (vs low) permeability gradually increased investment in individual mobility (permeability by time interaction effect: *F*(1, 437) = 21.76, *p* < .001, η^2^ = .050). H4b is not confirmed as there is no significant permeability by efficacy by time interaction effect on investment in individual mobility.

# III Extended results Study 1 and Study 2

## Demographics

Participants were recruited through the online platform Academic Prolific and they were UK residents with high approval rate in the platform. Participants were paid a minimum participation fee according to the guidelines of Academic Prolific.

In both studies, we employed a between-subjects design. In both studies, the gender ratio did not differ significantly across efficacy conditions (Study 1: χ^2^ (1, n = 232) = 0.01, p = .927; Study 2: χ^2^ (1, n = 664) = 0.16, p = .692), prospects-of-mobility conditions (Study 1: χ^2^ (1, n = 232) < 0.01, p = .998; Study 2: χ^2^ (1, n = 443) = 1.90, p = .692), and group permeability conditions (Study 2: χ^2^ (1, n = 441) = 0.51, p = .474). Moreover, participants’ age did not differ significantly across efficacy conditions (Study 1: t(229) = 0.88, p = .379; Study 2: t(660) = 0.28, p = .783), prospects-of-mobility conditions (Study 1: t(229) = 1.63, p = .104; Study 2: t(440) = 1.54, p = .124), and group permeability conditions (Study 2: t(437) = 1.34, p = .180).

**Table S1.** Basic demographic information per condition

|  | **Conditions** | **N** | **Women** | **Mean Age** | **Std. Dev. Age** |
| --- | --- | --- | --- | --- | --- |
| **Study 1** | **Low efficacy & Low prospects of mobility** | 57 | 38 | 33,35 | 11,881 |
|  | **High efficacy & Low prospects of mobility** | 62 | 41 | 31,08 | 10,010 |
|  | **Low efficacy & High prospects of mobility** | 57 | 38 | 30,02 | 10,755 |
|  | **High efficacy & High prospects of mobility** | 56 | 37 | 29,73 | 10,149 |
| **Study 2** | **Low efficacy & Low prospects of mobility & low permeability** | 110 | 40 | 31,35 | 10,560 |
|  | **High efficacy & Low prospects of mobility & low permeability** | 113 | 42 | 31,44 | 8,446 |
|  | **Low efficacy & High prospects of mobility & low permeability** | 110 | 48 | 31,96 | 10,867 |
|  | **High efficacy & High prospects of mobility & low permeability** | 110 | 47 | 33,89 | 11,800 |
|  | **Low efficacy & High prospects of mobility & high permeability** | 112 | 42 | 32,30 | 9,841 |
|  | **High efficacy & High prospects of mobility & high permeability** | 109 | 46 | 30,93 | 8,052 |

## Comparing effect sizes

To compare effect sizes, we converted the partial eta squared of our efficacy, prospects-of-mobility and group permeability effects to Cohen’s *d* and then we computed the 95% confidence intervals of each effect size. The calculation is based on the variance for the theoretical sampling distribution of Cohen’s *d*, and assumes normally distributed data, reasonable sample sizes, and values of *d* that are not very large [1]. Given that the efficacy effect emerged over time while the prospects-of-mobility effect was more or less stable over time, we focused on the size of the Efficacy by Time interaction effect and the size of the main prospects-of-mobility effect.

The estimated 95% confidence intervals of Cohen’s *d* revealed that the efficacy effect on the collective action is significantly larger than the prospects-of-mobility effect (in both studies) and the group permeability effect (in Study 2) on the same outcome variable (Tables S2-S3).

**Table S2.** Effect sizes of efficacy and prospects of mobility on investment in the three strategies (Study 1)

| **Outcome variables** | **Predictor variables** | **η^2^** | **Cohen's d** | **95% confidence intervals** | |
| --- | --- | --- | --- | --- | --- |
|  |  |  |  | **lower bound** | **upper bound** |
| **Redistribution** | **Efficacy by Time** | .130 | .77 | 0.51 | 1.04 |
|  | **Prospects of mobility** | .003 | .11 | -0.15 | 0.37 |
| **Individual Mobility** | **Efficacy by Time** | .050 | .46 | 0.20 | 0.72 |
|  | **Prospects of mobility** | .030 | .35 | 0.09 | 0.61 |
| **Inaction** | **Efficacy by Time** | .020 | .29 | 0.03 | 0.54 |
|  | **Prospects of mobility** | .010 | .20 | -0.06 | 0.46 |

**Table S3.** Effect sizes of efficacy, prospects of mobility, and group permeability on investment in the three strategies (Study 2)

| **Outcome variables** | **Predictor variables** | **η^2^** | **Cohen's d** | **95% confidence intervals** | |
| --- | --- | --- | --- | --- | --- |
|  |  |  |  | **lower bound** | **upper bound** |
| **Redistribution** | **Efficacy by Time** | .080 | .59 | 0.43 | 0.75 |
|  | **Prospects of mobility** | .006 | .16 | -0.03 | 0.34 |
|  | **Permeability by Time** | <.001 | .02 | -0.17 | 0.21 |
| **Individual Mobility** | **Efficacy by Time** | <.001 | .02 | -0.14 | 0.17 |
|  | **Prospects of mobility** | .050 | .46 | 0.27 | 0.66 |
|  | **Permeability by Time** | .050 | .46 | 0.27 | 0.65 |
| **Inaction** | **Efficacy by Time** | .070 | .55 | 0.39 | 0.70 |
|  | **Prospects of mobility** | .020 | .29 | 0.10 | 0.47 |
|  | **Permeability by Time** | .050 | .46 | 0.27 | 0.65 |

##

## Effects on beliefs

Previous theoretical and empirical work in social psychology [2,3] has shown that subjective perceptions of disadvantaged members of the society about (1) the legitimacy of economic inequality, (2) the inter-group permeability (perceptions about the likelihood of upward mobility), and (3) the efficacy of collective action determine whether the individual will adopt a social mobility strategy or will engage in collective action. Previous work has shown that increased legitimacy of an unequal distribution of resources reduces collective action against inequality [4]. Furthermore, increased belief in inter-group permeability will lead to the adoption of an individual mobility strategy at the cost of engaging in collective action [5,6]. In contrast, the belief in high efficacy of collective action leads to increased engagement in collective action and avoidance of an individual mobility strategy [7,8]. In the present study, at the end of the first and last round, we elicit beliefs regarding the fairness of initial income gap, the permeability between income group boundaries, and the efficacy of the collective action. Participants reported their beliefs on a 7-point Likert scale.

Overall, efficacy had no substantial impact on perceptions of fairness and permeability (Tables S4-S5 and Fig. S1). However, in Study 2 low efficacy led to decreased perceptions of fairness, especially in the last round. As expected, self-reported efficacy was lower in the low efficacy condition and this efficacy effect was more prominent in the last compared to the first round (Tables S4-S5 and Fig. S1).

On the other hand, exposure to high prospects of mobility increased the perceived likelihood of upward mobility in both rounds but had no impact on fairness perceptions or efficacy beliefs (Tables S4-S5 and Fig. S1). High permeability of income group boundaries substantially increased the perceived likelihood of upward mobility but only in the last round (Table S5 and Fig. S1). Interestingly, high group permeability also increased fairness perceptions in the last compared to the first round.

According to social identity theory, identification with the low-status group is a strong predictor of the willingness to engage in collective action to redress inequalities [3,9]. A relatively unaddressed question is to what extent the actual efficacy of collective action and the permeability of group boundaries could influence the strength of identification with low-status groups. To address this question, we also measured the extent to which participants identify with the low-income group at the end of the experiment. On average, low-income individuals identified moderately with their income group (Study 1: M = 3.91, SD = 1.90; Study 2: M = 3.76, SD = 1.77; on a 7-point Likert scale). In both studies, high efficacy substantially increased the strength of identification (Study 1: F(1,231) = 10.10, p = .002, η^2^=.042; Study 2: F(1,661) = 18.75, p < .001, η^2^=.028). On the other hand, exposure to high prospects of mobility and high group permeability did not influence strength of group identification.

**Table S4.** Three-way mixed ANOVA quantifying how perceived fairness of income gap, perceived permeability, and perceived efficacy differ across conditions and over time in Study 1.

| **Predictor variables** |  | **Outcome variables** | | | | | | | | |
| --- | --- | --- | --- | --- | --- | --- | --- | --- | --- | --- |
|  | **df** | **Fairness perceptions** | | | **Perceived permeability** | | | **Perceived efficacy** | | |
|  |  | **F** | **p** | **η^2^** | **F** | **p** | **η^2^** | **F** | **p** | **η^2^** |
| **Efficacy** | 1, 228 | 1.42^f^ | .235 | .006 | 2.25 | .135 | .010 | 37.50*^a^ | <.001 | .140 |
| **Prospects of mobility** | 1, 228 | 3.68^g^ | .056 | .020 | 19.50*^c^ | .016 | .080 | 0.37 | .545 | .002 |
| **Time** | 1, 228 | 20.39* | .010 | .080 | 127.13*^d^ | <.001 | .360 | 67.12* | <.001 | .230 |
| **Efficacy x Prospects of mobility** | 1, 228 | 0.73 | .394 | .003 | 2.63 | .107 | .010 | 0.37 | .545 | .002 |
| **Efficacy x Time** | 1, 228 | 1.86 | .174 | .008 | 5.00 | .026 | .020 | 50.48*^b^ | <.001 | .180 |
| **Prospects of mobility x Time** | 1, 228 | 0.07 | .795 | <.001 | 1.84^e^ | .177 | .008 | 0.08 | .771 | <.001 |
| **Efficacy x Prospects of mobility x Time** | 1, 228 | 0.20 | .653 | .001 | 1.64 | .201 | .007 | 4.65 | .032 | .020 |

**p* < .017 (p value adjusted for multiple testing)

^a^ Preregistered secondary hypothesis S1a was confirmed

^b^ Preregistered secondary hypothesis S1b was confirmed

^c^ Preregistered secondary hypothesis S2a was confirmed

^d^ Preregistered secondary hypothesis S2b was confirmed

^e^ Preregistered secondary hypothesis S2c was not confirmed

^f^ Preregistered secondary hypothesis S3a was not confirmed

^g^ Preregistered secondary hypothesis S3c was not confirmed

**Table S5.** Three-way mixed ANOVAs quantifying how perceived fairness of income gap, perceived permeability, and perceived efficacy differ across conditions and over time in Study 2.

| **Predictor variables** |  | **Outcome variables** | | | | | | | | |
| --- | --- | --- | --- | --- | --- | --- | --- | --- | --- | --- |
|  | **df** | **Fairness perceptions** | | | **Perceived permeability** | | | **Perceived efficacy** | | |
|  |  | **F** | **p** | **η^2^** | **F** | **p** | **η^2^** | **F** | **p** | **η^2^** |
| **Efficacy^1^** | 1, 437 | 4.70 | .031 | .010 | 4.04 | .045 | .009 | 49.38*^a^ | <.001 | .100 |
| **Prospects of mobility^1^** | 1, 437 | 0.05 | .828 | <.001 | 20.75*^c^ | <.001 | .050 | 0.57 | .452 | .001 |
| **Time^1^** | 1, 437 | 0.07 | .791 | <.001 | 199.97*^d^ | <.001 | .310 | 128.37* | <.001 | .230 |
| **Efficacy x Prospects of mobility^1^** | 1, 437 | 0.29 | .593 | <.001 | 0.02 | .865 | <.001 | 0.75 | .386 | .002 |
| **Efficacy x Time^1^** | 1, 437 | 14.60* | <.001 | .030 | 8.41* | .004 | .020 | 39.18*^b^ | <.001 | .080 |
| **Prospects of mobility x Time^1^** | 1, 437 | 0.07 | .791 | <.001 | 10.26*^e^ | .001 | .020 | 4.70 | .031 | .010 |
| **Efficacy x Prospects of mobility x Time^1^** | 1, 437 | 0.19 | .660 | <.001 | 0.91 | .341 | .002 | 0.82 | .366 | .002 |
| **Efficacy^2^** | 1, 437 | 7.77* | .005 | .020 | 5.86 | .016 | .010 | 63.92* | <.001 | .130 |
| **Permeability^2^** | 1, 437 | 1.15 | .285 | .003 | 47.27* | <.001 | .110 | 2.03 | .155 | .005 |
| **Time^2^** | 1, 437 | 5.95 | .015 | .010 | 47.61* | <.001 | .080 | 129.82* | <.001 | .230 |
| **Efficacy x Permeability^2^** | 1, 437 | 0.01 | .966 | <.001 | 1.79 | .181 | .010 | 0.12 | .725 | <.001 |
| **Efficacy x Time^2^** | 1, 437 | 22.15* | <.001 | .050 | 9.01* | .003 | .030 | 46.28* | <.001 | .100 |
| **Permeability x Time^2^** | 1, 437 | 5.95^g^ | .015 | .010 | 99.24*^f^ | <.001 | .160 | 6.42 | .012 | .010 |
| **Efficacy x Permeability x Time^2^** | 1, 437 | 0.29 | .588 | .001 | 0.55 | .456 | .004 | 1.63 | .202 | <.004 |

^1^ First ANOVA testing the effects of efficacy and prospects of mobility (only impermeable conditions were included)

^2^ Second ANOVA testing the effects of efficacy and group permeability (only high prospects-of mobility-conditions were included)

**p* < .008 (p value adjusted for multiple testing)

^a^ Secondary hypothesis S1a (first preregistration) was confirmed

^b^ Secondary hypothesis S1b (first preregistration) was confirmed

^c^ Secondary hypothesis S2a (first preregistration) was confirmed

^d^ Secondary hypothesis S2b (first preregistration) was confirmed

^e^ Secondary hypothesis S2c (first preregistration) was confirmed

^f^ Secondary hypothesis S1 (second preregistration) was confirmed

^g^ Secondary hypothesis S2 (second preregistration) was not confirmed

## Growth mixture model analysis

To identify distinct behavioral types, we fitted latent class linear mixed models (LCLMM) also known as growth mixture models [10,11]. The LCLMM assumes that the population is divided in a finite number of latent classes. Each latent class is characterized by a specific trajectory modelled by a class-specific linear mixed model. We performed both fixed (Latent Class Growth Analysis; LCGA) and random intercept or random intercept and slope (Growth Mixture Model; GMM) effects models.

We first fitted fixed-effects models (LCGA) with increasing numbers of classes and we compared the fit between each model to select the one that best describes the data. We repeated this procedure for random-effects models (random intercepts and random intercepts and random slopes; GMM). In both studies, we found that a parsimonious fixed-effect model with four (Study 1) and five (Study 2) classes showed better statistical fit to the data (using Bayesian Information Criteria) than the random-effects models (Tables S6-S7).

The analysis revealed four behavioral types in Study 1 and five behavioral types in Study 2. In both studies, we identified three generic types: “mobility optimists”, “egalitarians”, and “disillusioned” individuals. “Egalitarians” were further divided into a moderate and an extreme version. In Study 2, the “disillusioned” were also divided into a moderate and an extreme version.

As shown in Tables S8-S9, in both studies, the actual number of “egalitarians” was substantially lower than the expected number in the low efficacy condition. On the other hand, the actual number of “mobility optimists” in Study 1 and the number of “disillusioned” individuals in both studies was substantially higher than the expected number in the low efficacy condition. Exposure to high prospects of mobility led to increased number of “mobility optimists” (compared to expected number) but this increase did not reach statistical significance in both studies. Permeability of income group boundaries also had no significant effect in the prevalence of all behavioral types.

**Table S6.** Likelihood statistics and Bayesian Information Criteria for LCGA and GMM (Study 1)

| **Model** | **Classes** | **-2LL** | **df** | **BIC** |
| --- | --- | --- | --- | --- |
| **Fixed intercept and slope (LCGA)** | 1 | -18878.38 | 5 | 37784.0 |
|  | 2 | -18577.23 | 10 | 37208.9 |
|  | 3 | -18480.17 | 15 | 37042.0 |
|  | **4** | **-18460.11** | **20** | **37029.2** |
| **Random intercept (GMM)** | 1 | -18878.38 | 6 | 37789.5 |
|  | 2 | -18577.23 | 11 | 37214.4 |
|  | 3 | -18480.17 | 16 | 37047.5 |
|  | 4 | -18460.11 | 21 | 37034.6 |
| **Random intercept and slope (GMM)** | 1 | -18878.38 | 8 | 37800.3 |
|  | 2 | -18577.23 | 13 | 37225.3 |
|  | 3 | -18480.17 | 18 | 37058.4 |
|  | 4 | -18460.11 | 23 | 37045.5 |

**Table S7.** Likelihood statistics and Bayesian Information Criteria for LCGA and GMM (Study 2)

| **Model** | **Classes** | **-2LL** | **df** | **BIC** |
| --- | --- | --- | --- | --- |
| **Fixed intercept and slope (LCGA)** | 1 | -53830.29 | 6 | 107699.6 |
|  | 2 | -52375.96 | 11 | 104823.4 |
|  | 3 | -51944.30 | 16 | 103992.6 |
|  | 4 | -51853.22 | 21 | 103842.9 |
|  | **5** | **-51813.00** | **26** | **103795.0** |
| **Random intercept (GMM)** | 1 | -53830.29 | 7 | 107706.1 |
|  | 2 | -52375.96 | 12 | 104829.9 |
|  | 3 | -51944.30 | 17 | 103999.1 |
|  | 4 | -51856.42 | 22 | 103855.8 |
|  | 5 | -51822.36 | 27 | 103815.6 |
| **Random intercept and slope (GMM)** | 1 | -53830.29 | 9 | 107719.1 |
|  | 2 | -52375.96 | 14 | 104842.9 |
|  | 3 | -51944.30 | 19 | 104012.1 |
|  | 4 | -51856.42 | 24 | 103868.8 |
|  | 5 | -51822.36 | 29 | 103828.5 |

**Table S8.** Contingency table to compare behavioral types across conditions (Study 1)

| **Condition** | **Mobility Optimists** | **Moderate Egalitarians** | **Extreme Egalitarians** | **Disillusioned** |
| --- | --- | --- | --- | --- |
| **high efficacy** | 38 (55.4) | 57 (40.2) | 15 (9.7) | 8 (12.7) |
| **low efficacy** | 71 (53.6) | 22 (38.8) | 4 (9.3) | 17 (12.3) |
| **high prospects of mobility** | 60 (53.1) | 38 (38.5) | 7 (9.3) | 8 (12.2) |
| **low prospects of mobility** | 49 (55.9) | 41 (40.5) | 12 (9.7) | 17 (12.8) |

Values indicate the actual number of low-income members exhibiting a particular behavioral type and values in brackets depict the expected number.

**Table S9.** Contingency table to compare behavioral types across conditions (Study 2)

| **Condition** | **Mobility Optimists** | **Moderate Egalitarians** | **Extreme Egalitarians** | **Moderate Disillusioned** | **Extreme Disillusioned** |
| --- | --- | --- | --- | --- | --- |
| **high efficacy** | 127 (121.5) | 117 (79) | 31 (22) | 32 (69.5) | 25 (40) |
| **low efficacy** | 116 (121.5) | 41 (79) | 13 (22) | 107 (69.5) | 55 (40) |
| **high prospects of mobility** | 85 (75) | 50 (50.2) | 11 (15.4) | 47 (47.7) | 27 (31.8) |
| **low prospects of mobility** | 66 (76) | 51 (50.8) | 20 (15.6) | 49 (48.3) | 37 (32.2) |
| **low permeability** | 85 (88.3) | 50 (53.4) | 11 (12) | 47 (44.9) | 27 (21.5) |
| **high permeability** | 92 (88.7) | 57 (53.6) | 13 (12) | 43 (45.1) | 16 (21.5) |

Values indicate the actual number of low-income members exhibiting a particular behavioral type and values in brackets depict the expected number.

# IV Additional and exploratory analyses

## Robustness test: Testing the effect of covariates

To eliminate potential age-specific and gender-specific effects across our conditions and to control for participants’ performance in the real-effort task (i.e., Counting Zeros Task), we performed three-way mixed ANCOVAs using the aforementioned variables as covariates. In both studies, controlling for the effect of these variables on our outcome variable did not influence main and interaction effects between our conditions and time (Tables S10-S11). Gender, and task performance had no influence on our outcome variables. In Study 1, age correlated positively with collective action.

**Table S10.** Three-way mixed ANOVAs using age, gender, and performance in the real-effort task as covariates to quantify how investment in three strategies differs across conditions in Study 1.

| **Predictor variables** |  | **Outcome variables** | | | | | | | | |
| --- | --- | --- | --- | --- | --- | --- | --- | --- | --- | --- |
|  | **df** | **Redistribution** | | | **Mobility** | | | **Inaction** | | |
|  |  | **F** | **p** | **η^2^** | **F** | **p** | **η^2^** | **F** | **p** | **η^2^** |
| **Efficacy** | 1, 225 | 3.51 | .062 | .020 | 0.70 | .402 | .003 | 0.76 | .384 | .003 |
| **prospects of mobility** | 1, 225 | 0.22 | .639 | .001 | 6.16* | .013 | .030 | 4.19 | .042 | .020 |
| **Time** | 1, 227 | 14.52* | <.001 | .060 | 2.50 | .115 | .010 | 4.90 | .028 | .020 |
| **Age** | 1, 224 | 8.52* | .004 | .040 | 2.16 | .143 | .010 | 3.17 | .076 | .010 |
| **Sex** | 1, 224 | 2.68 | .103 | .010 | 2.60 | .108 | .010 | 0.06 | .804 | <.001 |
| **Task Performance** | 1, 224 | 0.00 | .957 | <.001 | 0.02 | .894 | <.001 | 0.10 | .754 | <.001 |
| **Efficacy x prospects of mobility** | 1, 225 | 0.19 | .663 | .001 | 0.18 | .672 | .001 | 0.09 | .769 | <.001 |
| **Efficacy x Time** | 1, 227 | 34.35* | <.001 | .130 | 10.45* | .001 | .040 | 5.72 | .018 | .020 |
| **prospects of mobility x Time** | 1, 227 | 0.09 | .769 | <.001 | 0.10 | .747 | <.001 | 0.12 | .727 | .001 |
| **Efficacy x prospects of mobility x Time** | 1, 227 | 0.22 | .641 | .001 | 0.41 | .522 | .002 | 0.73 | .394 | .003 |

**p* < .017 (p value adjusted for multiple testing)

**Table S11.** Three-way mixed ANOVAs using age, gender, and performance in the real-effort task as covariates to quantify how investment in three strategies differs across conditions in Study 2.

| **Predictor variables** |  | **Outcome variables** | | | | | | | | |
| --- | --- | --- | --- | --- | --- | --- | --- | --- | --- | --- |
|  | **df** | **Redistribution** | | | **Mobility** | | | **Inaction** | | |
|  |  | **F** | **p** | **η^2^** | **F** | **p** | **η^2^** | **F** | **p** | **η^2^** |
| **Efficacy^1^** | 1, 436 | 12.48* | <.001 | .030 | 9.66* | .002 | .020 | 1.05 | .306 | .002 |
| **Prospects of mobility^1^** | 1, 436 | 3.00 | .084 | .007 | 24.20* | <.001 | .050 | 8.13* | .004 | .020 |
| **Time^1^** | 1, 436 | 147.62* | <.001 | .250 | 18.05* | <.001 | .040 | 236.16* | <.001 | .350 |
| **Age^1^** | 1, 433 | 0.44 | .508 | .001 | 0.36 | .546 | .001 | 1.00 | .317 | .002 |
| **Sex^1^** | 1, 433 | 1.26 | .263 | .003 | 0.31 | .576 | .001 | 1.27 | .260 | .003 |
| **Task Performance^1^** | 1, 433 | 2.17 | .142 | .005 | 0.44 | .509 | .001 | 1.47 | .225 | .003 |
| **Efficacy x Prospects of mobility^1^** | 1, 435 | 0.08 | .773 | <.001 | 0.02 | .879 | <.001 | 0.04 | .843 | <.001 |
| **Efficacy x Time^1^** | 1, 436 | 21.86* | .004 | .050 | 3.27 | .071 | .007 | 36.99* | <.001 | .080 |
| **Prospects of mobility x Time^1^** | 1, 436 | 0.63 | .428 | .001 | 0.04 | .835 | <.001 | 0.88 | .350 | .002 |
| **Efficacy x Prospects of mobility x Time^1^** | 1, 436 | 0.12 | .728 | <.001 | 1.48 | .225 | .003 | 2.25 | .134 | .005 |
| **Efficacy^2^** | 1, 434 | 9.60* | .002 | .020 | 2.18 | .140 | .003 | 5.09 | .025 | .010 |
| **Permeability^2^** | 1, 435 | 0.37 | .540 | .001 | 1.65 | .200 | .008 | 0.14 | .705 | <.001 |
| **Time^2^** | 1, 435 | 177.12* | <.001 | .290 | 0.01 | .914 | <.001 | 164.35* | <.001 | .270 |
| **Age^2^** | 1, 432 | 3.91 | .049 | .009 | <0.01 | .991 | <.001 | 5.97 | .015 | .010 |
| **Sex^2^** | 1, 432 | <0.01 | .967 | <.001 | <0.01 | .985 | <.001 | 0.14 | .712 | <.001 |
| **Task Performance^2^** | 1, 432 | 0.07 | .798 | <.001 | 1.24 | .267 | .003 | 0.98 | .322 | .002 |
| **Efficacy x Permeability^2^** | 1, 434 | 0.42 | .815 | .001 | 2.33 | .127 | .007 | 1.46 | .228 | .003 |
| **Efficacy x Time^2^** | 1, 435 | 45.66* | <0.001 | .100 | 1.64 | .201 | .004 | 23.81* | .001 | .050 |
| **Permeability x Time^2^** | 1, 435 | 0.05 | .815 | <.001 | 21.15* | .006 | .050 | 21.36* | .005 | .050 |
| **Efficacy x Permeability x Time^2^** | 1, 435 | 4.97 | .026 | .010 | 3.53 | .061 | .008 | <0.01 | .982 | <.001 |

^1^ ANOVA testing the effects of efficacy and prospects of mobility (only low permeability conditions were included)

^2^ ANOVA testing the effects of efficacy and group permeability (only high prospects-of-mobility conditions were included)

**p* < .008 (p value adjusted for multiple testing)

##

## Feedback effects on collective action

High redistribution thresholds and thus low efficacy of disadvantaged individuals gradually crowded out collective action, while high efficacy led to a more stable collective action over time. This time-dependent efficacy effect is probably driven by the substantially different feedback about the success of the redistribution strategy that disadvantaged individuals received in the two efficacy conditions.

We performed a hierarchical regression analysis to test whether feedback about redistribution outcomes in the current round influence collective action in the next round. In the first step of the regression, we controlled for the collective action in the current round. In the second step, we introduced the feedback about redistribution outcomes (i.e., no inequality reduction, reduced inequality, or absolute equality) as our main indicator. In both studies, analysis confirmed our hypothesis that positive feedback about redistribution outcomes increases collective action in the next round (Tables S12-S13).

**Table S12**. Series of hierarchical regressions quantifying the effect of feedback about redistribution outcome on collective action in the next round (Study 1).

| **Predictor variables** | **B** | **SE B** | **β** | ***t*** |
| --- | --- | --- | --- | --- |
| **Redistribution Outcome (Round 1)^1^** | 0.64 | 0.27 | .14 | 2.36* |
| **Redistribution Outcome (Round 2)^2^** | 0.80 | 0.31 | .16 | 2.56* |
| **Redistribution Outcome (Round 3)^3^** | 0.68 | 0.32 | .14 | 2.10* |
| **Redistribution Outcome (Round 4)^4^** | 0.77 | 0.31 | .16 | 2.48* |
| **Redistribution Outcome (Round 5)^5^** | 0.67 | 0.37 | .12 | 1.81 |
| **Redistribution Outcome (Round 6)^6^** | 1.26 | 0.29 | .26 | 4.31*** |
| **Redistribution Outcome (Round 7)^7^** | 0.28 | 0.32 | .06 | 0.89 |
| **Redistribution Outcome (Round 8)^8^** | 1.31 | 0.30 | .27 | 4.43*** |
| **Redistribution Outcome (Round 9)^9^** | 0.81 | 0.30 | .17 | 2.67** |

Note: The table presents only the second step of the hierarchical regression and omits the collective action in the current round which was included in the first step of the regression.

**p* < .05, ***p* < .01, ****p* < .001

^1^ Outcome variable is collective action in Round 2

^2^ Outcome variable is collective action in Round 3

^3^ Outcome variable is collective action in Round 4

^4^ Outcome variable is collective action in Round 5

^5^ Outcome variable is collective action in Round 6

^6^ Outcome variable is collective action in Round 7

^7^ Outcome variable is collective action in Round 8

^8^ Outcome variable is collective action in Round 9

^9^ Outcome variable is collective action in Round 10

**Table S13**. Series of hierarchical regressions quantifying the effect of feedback about redistribution outcome on collective action in the next round (Study 2).

| **Predictor variables** | **B** | **SE B** | **β** | ***t*** |
| --- | --- | --- | --- | --- |
| **Redistribution Outcome (Round 1)^1^** | 0.56 | 0.14 | .14 | 3.91*** |
| **Redistribution Outcome (Round 2)^2^** | 0.61 | 0.15 | .14 | 4.03*** |
| **Redistribution Outcome (Round 3)^3^** | 0.76 | 0.16 | .17 | 4.74*** |
| **Redistribution Outcome (Round 4)^4^** | 0.91 | 0.16 | .19 | 5.52*** |
| **Redistribution Outcome (Round 5)^5^** | 0.30 | 0.17 | .07 | 1.79 |
| **Redistribution Outcome (Round 6)^6^** | 0.72 | 0.15 | .16 | 4.78*** |
| **Redistribution Outcome (Round 7)^7^** | 0.62 | 0.14 | .14 | 4.27*** |
| **Redistribution Outcome (Round 8)^8^** | 0.66 | 0.16 | .15 | 4.22*** |
| **Redistribution Outcome (Round 9)^9^** | 0.82 | 0.15 | .18 | 5.39*** |

Note: The table presents only the second step of the hierarchical regression and omits the collective action in the current round which was included in the first step of the regression.

**p* < .05, ***p* < .01, ****p* < .001

^1^ Outcome variable is collective action in Round 2

^2^ Outcome variable is collective action in Round 3

^3^ Outcome variable is collective action in Round 4

^4^ Outcome variable is collective action in Round 5

^5^ Outcome variable is collective action in Round 6

^6^ Outcome variable is collective action in Round 7

^7^ Outcome variable is collective action in Round 8

^8^ Outcome variable is collective action in Round 9

^9^ Outcome variable is collective action in Round 10

## Experiencing vs witnessing upward mobility

In the two low permeability conditions of Study 2 (low prospects of mobility & low permeability and high prospects of mobility & low permeability) upward mobility took place only in the first round and in subsequent rounds group boundaries were impermeable. In the first round, the two low-income members who allocated the most APs to the individual mobility strategy achieved upward mobility and the other two low-income members witnessed the upward mobility of the other two members. We tested whether experiencing or witnessing upward mobility influences investment in the three strategies in subsequent rounds (Round 2 to 10). We thus introduced a binary variable of personal mobility experience (named “Experience” in Table S17) in a four-way mixed ANOVA.

The test revealed that personal experience of upward mobility in the first round has no substantial impact on investment in redistribution or inaction (Table S14). Compared to witnessing the upward mobility of others, personal mobility experience increased investment in individual mobility. This effect may indicate an effect of personal experience of mobility but may also reflect the biased selection of participants who invested more in in individual mobility in the first round (i.e., autocorrelation effect). Nonetheless, personal mobility experience did not interact with time or any of our predictor variables (Table S14). Hence, we ignored this distinction when we examined main and interaction effects in the entire sample (i.e., collapsing participants who experienced and those who witnessed upward mobility).

**Table S14.** Four-way mixed ANOVAs quantifying how personal vs vicarious experience of upward mobility in the first round interacts with other predictor variables to affect investment in three strategies in Study 2

| **Predictor variables** |  | **Outcome variables** | | | | | | | |  |
| --- | --- | --- | --- | --- | --- | --- | --- | --- | --- | --- |
|  |  | **Redistribution** | | | **Mobility** | | | **Inaction** | | |
|  | **df** | **F** | **p** | **η^2^** | **F** | **p** | **η^2^** | **F** | **p** | **η^2^** |
| **Efficacy** | 1, 434 | 15.75 | .085 | .040 | 17.41 | .036 | .040 | 0.05 | .828 | <.001 |
| **Prospects of mobility** | 1, 434 | 3.44 | .064 | .008 | 30.57* | <.001 | .070 | 11.22* | .001 | .030 |
| **Time** | 1, 434 | 90.85* | <.001 | .170 | 7.41 | .007 | .020 | 139.46* | <.001 | .240 |
| **Experience** | 1, 434 | 2.27 | .133 | .005 | 10.06* | .002 | .020 | 2.25 | .135 | .005 |
| **Efficacy x Prospects of mobility** | 1, 434 | 0.01 | .917 | <.001 | 0.25 | .621 | .001 | 0.31 | .576 | .001 |
| **Efficacy x Experience** | 1, 434 | 0.05 | .830 | <.001 | 0.42 | .518 | .001 | 0.19 | .662 | <.001 |
| **Prospects of mobility x Experience** | 1, 434 | 0.39 | .534 | .001 | 0.25 | .615 | .001 | 0.02 | .881 | <.001 |
| **Efficacy x Prospects of mobility x Experience** | 1, 434 | 0.23 | .635 | .001 | 0.08 | .781 | <.001 | 0.04 | .836 | <.001 |
| **Efficacy x Time** | 1, 434 | 4.63 | .032 | .010 | 8.86* | .003 | .020 | 25.72* | .001 | .060 |
| **Prospects of mobility x Time** | 1, 434 | 0.00 | .957 | <.001 | 3.17 | .076 | .007 | 2.92 | .088 | .007 |
| **Experience x Time** | 1, 434 | 0.69 | .406 | .002 | 0.82 | .366 | .002 | 0.02 | .875 | <.001 |
| **Efficacy x Prospects of mobility x Time** | 1, 434 | 0.00 | .950 | <.001 | 0.19 | .660 | <.001 | 0.18 | .675 | <.001 |
| **Efficacy x Experience x Time** | 1, 434 | 0.63 | .429 | .001 | 0.32 | .572 | .001 | 0.02 | .885 | <.001 |
| **Prospects of mobility x Experience x Time** | 1, 434 | 4.58 | .033 | .010 | 3.21 | .074 | .007 | 0.03 | .852 | <.001 |
| **Efficacy x Prospects of mobility x Experience x Time** | 1, 434 | 0.01 | .942 | <.001 | 0.13 | .722 | <.001 | 0.21 | .644 | <.001 |

**p* < .008 (p value adjusted for multiple testing)

# Figure S1

**
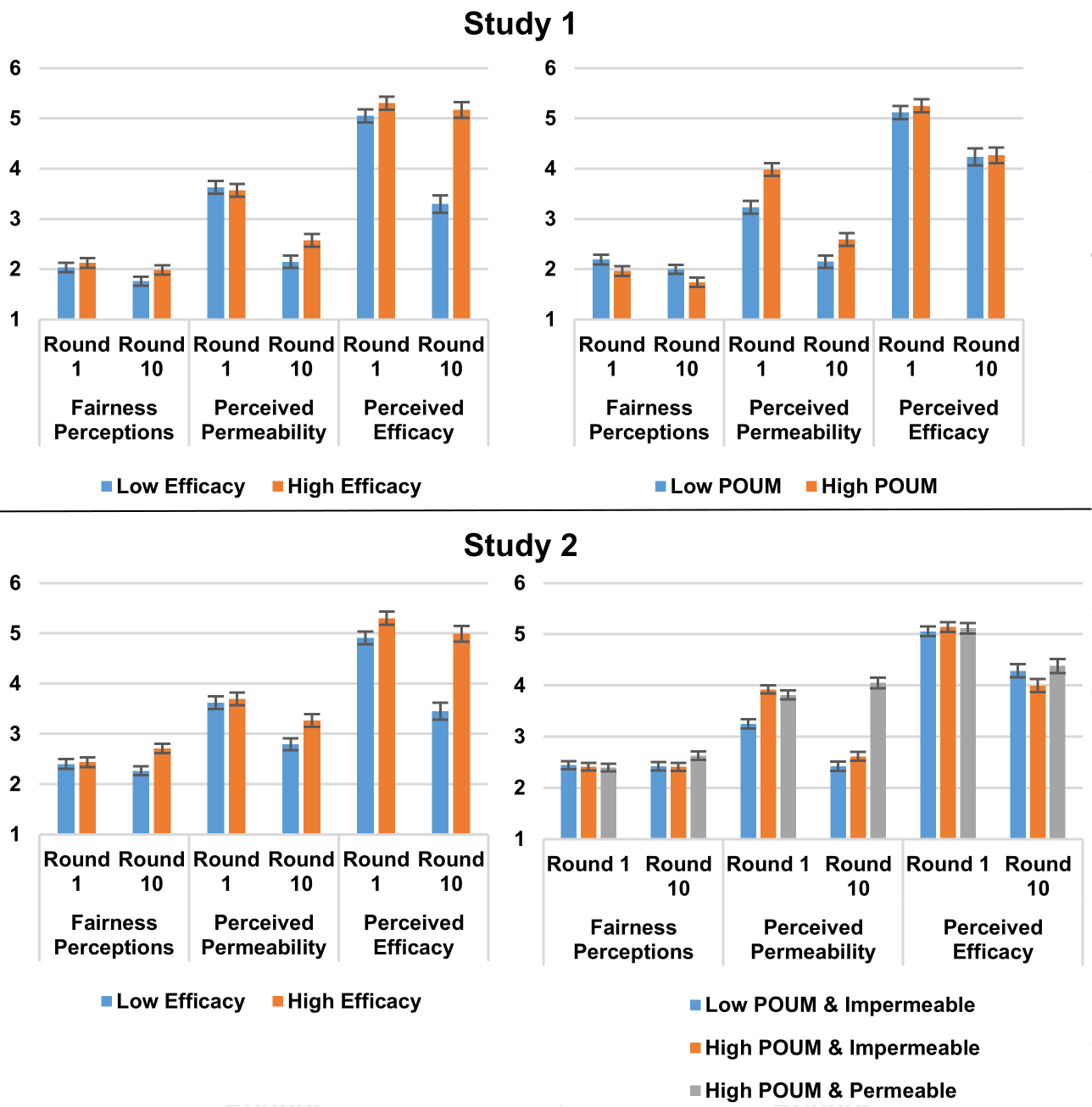
**

**Figure S1.** Efficacy, prospects of mobility and group permeability effects on beliefs. In both studies, perceptions of fairness, perceived permeability, and perceived efficacy were assessed at the end of the first and the last round. The upper part of the figure presents the results of Study 1 and the lower part the results of Study 2. As expected, low efficacy (right part of the figure) reduced perceived efficacy and led to increased fairness perception, but this effect hold true only in Study 2 (right lower part). Exposure to high prospects of mobility increased perceptions of permeability (left part of the figure). High permeability of income group boundaries also increased perceived permeability but only in the last round (left lower part). Moreover, high group permeability increased fairness perceptions in the last round (Left lower part).

# SI References

1 Gibbons, R. D., Hedeker, D. R. & Davis, J. M. Estimation of Effect Size from a Series of Experiments Involving Paired Comparisons. *J Educ Stat* **18**, 271-279 (1993). <https://doi.org:Doi> 10.3102/10769986018003271

2 Hornsey, M. J. Social identity theory and self‐categorization theory: A historical review. *Soc Personal Psychol* **2**, 204-222 (2008).

3 Jetten, J. *et al.* Consequences of Economic Inequality for the Social and Political Vitality of Society: A Social Identity Analysis. *Polit Psychol* **42**, 241-266 (2021). <https://doi.org:10.1111/pops.12800>

4 Ellemers, N., Wilke, H. & Vanknippenberg, A. Effects of the Legitimacy of Low Group or Individual Status on Individual and Collective Status-Enhancement Strategies. *Journal of personality and social psychology* **64**, 766-778 (1993). <https://doi.org:Doi> 10.1037/0022-3514.64.5.766

5 Ellemers, N., Vanknippenberg, A. & Wilke, H. The Influence of Permeability of Group Boundaries and Stability of Group Status on Strategies of Individual Mobility and Social-Change. *Brit J Soc Psychol* **29**, 233-246 (1990). <https://doi.org:DOI> 10.1111/j.2044-8309.1990.tb00902.x

6 Wright, S. C. Ambiguity, social influence, and collective action: Generating collective protest in response to tokenism. *Pers Soc Psychol B* **23**, 1277-1290 (1997). <https://doi.org:Doi> 10.1177/01461672972312005

7 van Zomeren, M., Saguy, T. & Schellhaas, F. M. H. Believing in "making a difference" to collective efforts: Participative efficacy beliefs as a unique predictor of collective action. *Group Process Interg* **16**, 618-634 (2013). <https://doi.org:10.1177/1368430212467476>

8 van Zomeren, M., Spears, R., Fischer, A. H. & Leach, C. W. Put your money where your mouth is! Explaining collective action tendencies through group-based anger and group efficacy. *Journal of personality and social psychology* **87**, 649-664 (2004). <https://doi.org:10.1037/0022-3514.87.5.649>

9 van Zomeren, M., Postmes, T. & Spears, R. Toward an integrative social identity model of collective action: A quantitative research synthesis of three socio-psychological perspectives. *Psychol Bull* **134**, 504-535 (2008). <https://doi.org:10.1037/0033-2909.134.4.504>

10 Proust-Lima, C., Philipps, V. & Liquet, B. Estimation of extended mixed models using latent classes and latent processes: the R package lcmm. *arXiv preprint arXiv:1503.00890* (2015).

11 Wardenaar, K. Latent Class Growth Analysis and Growth Mixture Modeling using R: A tutorial for two R-packages and a comparison with Mplus. (2020).
